# Transcriptomic analysis of human brain microvascular endothelial cells exposed to laminin binding protein (adhesion lipoprotein) and *Streptococcus pneumoniae*

**DOI:** 10.1038/s41598-021-87021-4

**Published:** 2021-04-12

**Authors:** Irene Jiménez-Munguía, Zuzana Tomečková, Evelína Mochnáčová, Katarína Bhide, Petra Majerová, Mangesh Bhide

**Affiliations:** 1 Laboratory of Biomedical Microbiology and Immunology, University of Veterinary Medicine and Pharmacy in Kosice, Komenskeho 73, Kosice, 04181 Slovak Republic; 2 Institute of Neuroimmunology of Slovak Academy of Sciences, Bratislava, Slovak Republic

**Keywords:** Microbiology, Pathogens, Transcriptomics, Blood-brain barrier, Diseases of the nervous system

## Abstract

*Streptococcus pneumoniae* invades the CNS and triggers a strong cellular response. To date, signaling events that occur in the human brain microvascular endothelial cells (hBMECs), in response to pneumococci or its surface adhesins are not mapped comprehensively. We evaluated the response of hBMECs to the adhesion lipoprotein (a laminin binding protein—Lbp) or live pneumococci. Lbp is a surface adhesin recently identified as a potential ligand, which binds to the hBMECs. Transcriptomic analysis was performed by RNA-seq of three independent biological replicates and validated with qRT-PCR using 11 genes. In total 350 differentially expressed genes (DEGs) were identified after infection with *S. pneumoniae,* whereas 443 DEGs when challenged with Lbp. Total 231 DEGs were common in both treatments. Integrative functional analysis revealed participation of DEGs in cytokine, chemokine, TNF signaling pathways and phagosome formation. Moreover, Lbp induced cell senescence and breakdown, and remodeling of ECM. This is the first report which maps complete picture of cell signaling events in the hBMECs triggered against *S. pneumoniae* and Lbp. The data obtained here could contribute in a better understanding of the invasion of pneumococci across BBB and underscores role of Lbp adhesin in evoking the gene expression in neurovascular unit.

## Introduction

*Streptococcus pneumoniae* (also known as pneumococcus) is a life-threatening pathogen responsible for high morbidity and mortality rates worldwide^1^. It can cross the blood–brain barrier (BBB) and cause meningitis, commonly known as pneumococcal meningitis, a rare but life-threatening medical emergency. Pneumococcus traverses the epithelial barrier via transcellular route or by disruption of the interepithelial tight junctions, however very little is known about molecular events occur during the penetration of the BBB^2^. The human BBB (a neurovascular unit) is composed of glia, astrocytes, pericytes and the brain microvascular endothelial cells (hBMECs)^3^. Being the interface between nervous tissue and the blood, hBMECs form the first cell line of contact with the circulating neuroinvasive pathogens^4^.

To date, the overall comprehension of the molecular mechanisms activated during endothelial cell invasion is poor. Receptor-mediated binding facilitates translocation of *S. pneumoniae*^5^. Recent study suggest that the platelet-activating factor receptor (PAFR) plays an important role in the adhesion of *S. pneumoniae* to endothelial cells, even though pneumococci do not directly bind PAFR^6^. The Laminin Receptor (LR) has also been proposed to mediate binding of *S. pneumoniae* to the host cells via its choline-binding protein A (CbpA), which may facilitate bacterial internalization^7,8^.

Several surface proteins of *S. pneumoniae* actively participate in the bacterial invasion. The previous report has shown that the surface-anchored neuraminidase A (NanA) promotes pneumococcal invasion of the brain endothelial cells^9^. Similarly, pneumolysin and choline-binding protein A (CbpA) are reported as important proteins for the development of the invasive pneumococcal meningitis^10^. The RlrA pilus has been shown to enhance the ability of pneumococci to adhere to the host cells^11,12^. Recently, using the high-throughput proteomic approach surface ligands of *S. pneumoniae*, which bind to the hBMECs were identified^13^. Among those surface ligands, adhesion lipoprotein (a laminin binding protein Lbp, Locus—Spr0906) was the promising candidate showing the highest binding ability to hBMECs. Lbp is also described as metal ion (zinc) binding protein (GO – molecular function), and its involvement in cell adhesion and metal ion transportation (GO – biological process) has been reported in other *Streptococcus* species including *S. agalactiae*^14,15^. Additionally, laminin-binding protein Spr0906 may play a critical role during pneumococcal invasion as occurs with homologous laminin-binding proteins participating in other bacterial infections^16,17^. The attachment of *S. agalactiae* to the hBMECs is mediated by the Laminin binding protein Lmb^14,17^. Laminin is one of the major components of basement membrane involved in the maintenance of cellular organization^14^. Hence, binding of the pneumococcal Lbp may facilitate the bacterial colonization and promote the bacterial invasion. It is noteworthy that, in *S. pneumoniae* most of the lipoproteins are individually dispensable*,* except Lbp, which might be one of the essential proteins for invading cell barriers as occurs with *S. agalactiae*^17^. Cell signaling events evoked by this protein has not been studied so far. Thus, we sought to evaluate the cell response triggered by Lbp in hBMECs.

Neuroinvasive bacteria are known to exploit multiple surface proteins to interact with hBMECs and evoke cell signaling events, which facilitate adhesion of pathogen on the endothelial surface and subsequently trigger the translocation (reviewed in^18,19^). For example, signaling events are directed to form docking structures for pathogens on the endothelial cells^20,21^, induce uptake and transcytosis^22–24^, upregulate proteases^25–27^, evoke apoptosis and anoikis^28^, relocate the cell junctional proteins and thus weaken the tight junction^29,30^, reorganization endothelial cytoskeleton (reviewed in^31^) and reorganize extracellular matrix^32^. Pneumococci, in particular, can induce inflammatory molecules such as cytokines and chemokines in the cells of the neurovascular unit. As a consequence, polymorphonuclear cells are attracted and activated to generate oxidative stress by the production of reactive oxygen species (ROS). Subsequent events lead to lipid peroxidation, mitochondrial damage and breaching of the BBB^2^. It is known that the immune response in pneumococcal meningitis is enhanced by TNF-α, which leads to the activation of NF-κB to regulate pro-inflammatory mediators such as IL-1β, IL-6, IFN- γ and chemokines^2^.

In the present study, we attempted to elucidate a complete picture of the cell signaling events evoked by adhesion lipoprotein Lbp in hBMECs. In parallel, the transcriptome of hBMECs induced by live *S. pneumoniae* was mapped. High-throughput RNA sequencing (RNA-seq), downstream detailed bioinformatic analysis and validation with qRT-PCR were performed.

## Results

### Recombinant adhesion lipoprotein Lbp used to challenge hBMECs

In order to understand the molecular events in hBMECs occurring due to the adhesion of pneumococcal Lbp, recombinant Lbp was overexpressed in *E. coli* and purified. Purity of the protein judged by SDS-PAGE and molecular mass measured with MALDI-TOF/MS are presented in Supplementary information Fig. S1.

### RNA-seq analysis

Gene expression in the challenged hBMECs (Lbp or *S. pneumoniae*) was evaluated by RNA-seq. Initial quality checks for RNA isolated from induced-hBMECs showed no sign of degradation (Supplementary information Fig. S2), while all cDNA libraries had optimal fragment size between 150 and 300 nt (Supplementary information Fig. S3). Total 9 cDNA libraries were generated from 3 biological replicates as follows: non-induced hBMECs (NC1 to NC3), hBMECs induced with *S. pneumoniae* (SP1 to SP3) or hBMECs induced with recombinant adhesion lipoprotein (Lbp-1 to Lbp-3). hBMECs exposed to recombinant Lbp yielded an average raw reads of 1.35 × 10^7^, while 1.52 × 10^7^ raw reads per sample were obtained for hBMECs induced with *S. pneumoniae* (Supplementary information Fig. S4). In total, 11,398 genes for each sample were mapped (Supplementary dataset 1.1 and 1.2).

### Differentially expressed genes (DEGSs) and validation

**Figure 1:**
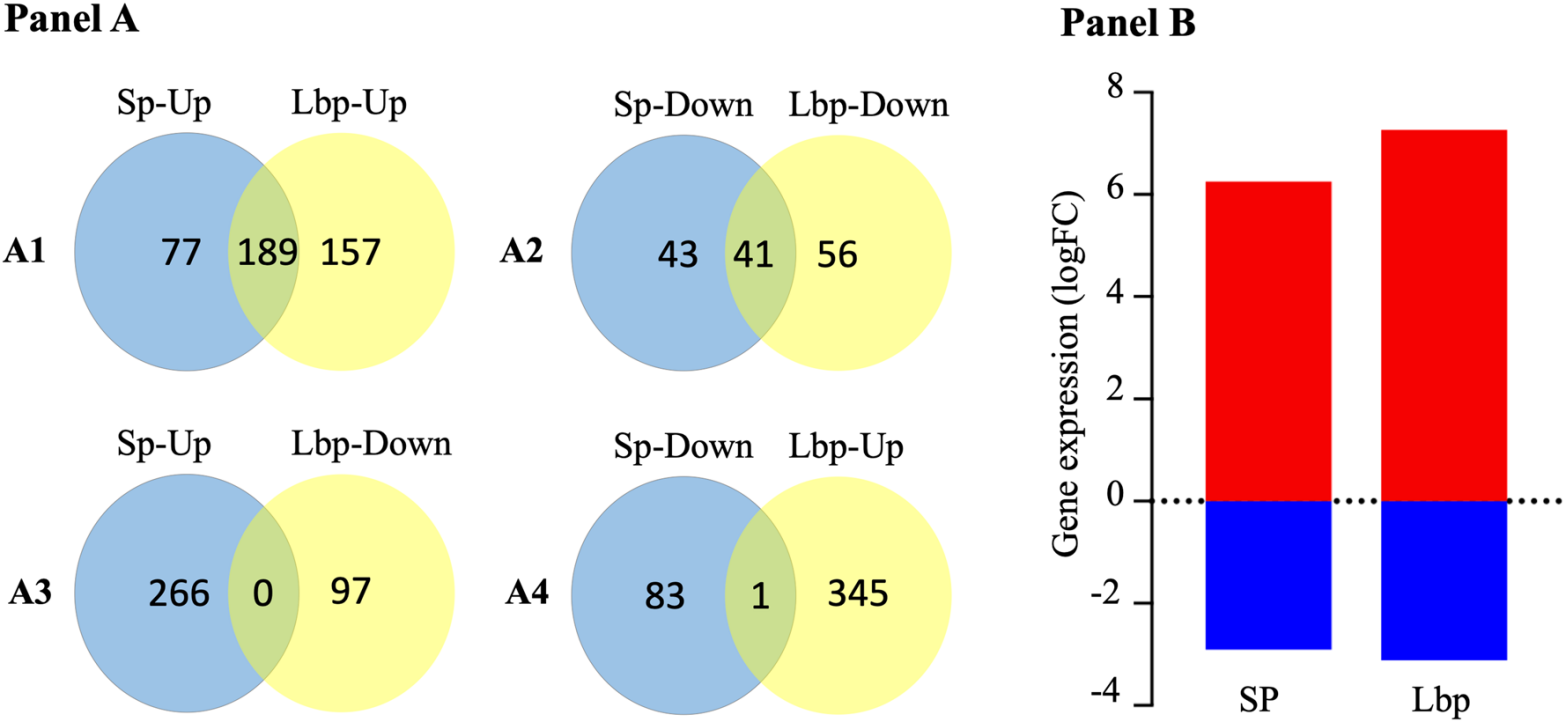
(Panel **A**) Differentially expressed genes (DEGs) graphically represented. Venn diagrams display number of DEGs identified by RNA-seq after incubation of hBMECs with *Streptococcus pneumoniae* (SP) and its adhesion lipoprotein (Lbp). DEGs in hBMECs incubated either with *SP* or Lbp are displayed in panels A1-A4. Up indicates upregulated genes and Down represents downregulated genes*.* Yellow and blue ellipses represent up- and down-regulated DEGs as indicated. Common DEGs in both treatments are shown as the intersection of ellipses. (Panel **B**) LogFC values of DEGs of induced hBMECs. Minimum and maximum log_2_ fold change values (LogFC) of DEGs identified in hBMECs after induction with *S. pneumoniae* (SP) or Lbp are presenting in columns. Red colour indicates up-regulation and blue color down-regulation.

Data analysis revealed a total of 443 DEGs (346 up-regulated genes and 97 down-regulated) for hBMECs induced with Lbp and 350 differentially expressed genes (DEGs) (266 up-regulated genes and 84 down-regulated) for hBMECs exposed to *S. pneumoniae* (Fig. 1A1–A4; Supplementary datasets 1.3 and 1.4). The log_2_ fold change values (LogFC), resulting from averaging three independent biological replicates for each condition, ranged between 6.25 and − 2.92 for hBMECs infected with *S. pneumoniae* and 7.26 to − 3.13 for hBMECs induced with Lbp (Fig. 1B). We noticed a consistent differential expression in most of the DEGs identified from both conditions. Genes expression changed in the same trend, except one gene (*btc*) which was up-regulated (LogFC = 2.53) in hBMECs induced with Lbp and down-regulated (LogFC = − 1.56) in *S. pneumoniae* infected cells (Fig. 1A4). A total of 231 DEGs were observed to be common in both treatments (189 up-regulated, 41 down-regulated and gen *btc* mentioned above) (Fig. 1A1,A2,A4; Supplementary dataset 1.5).

**Figure 2:**
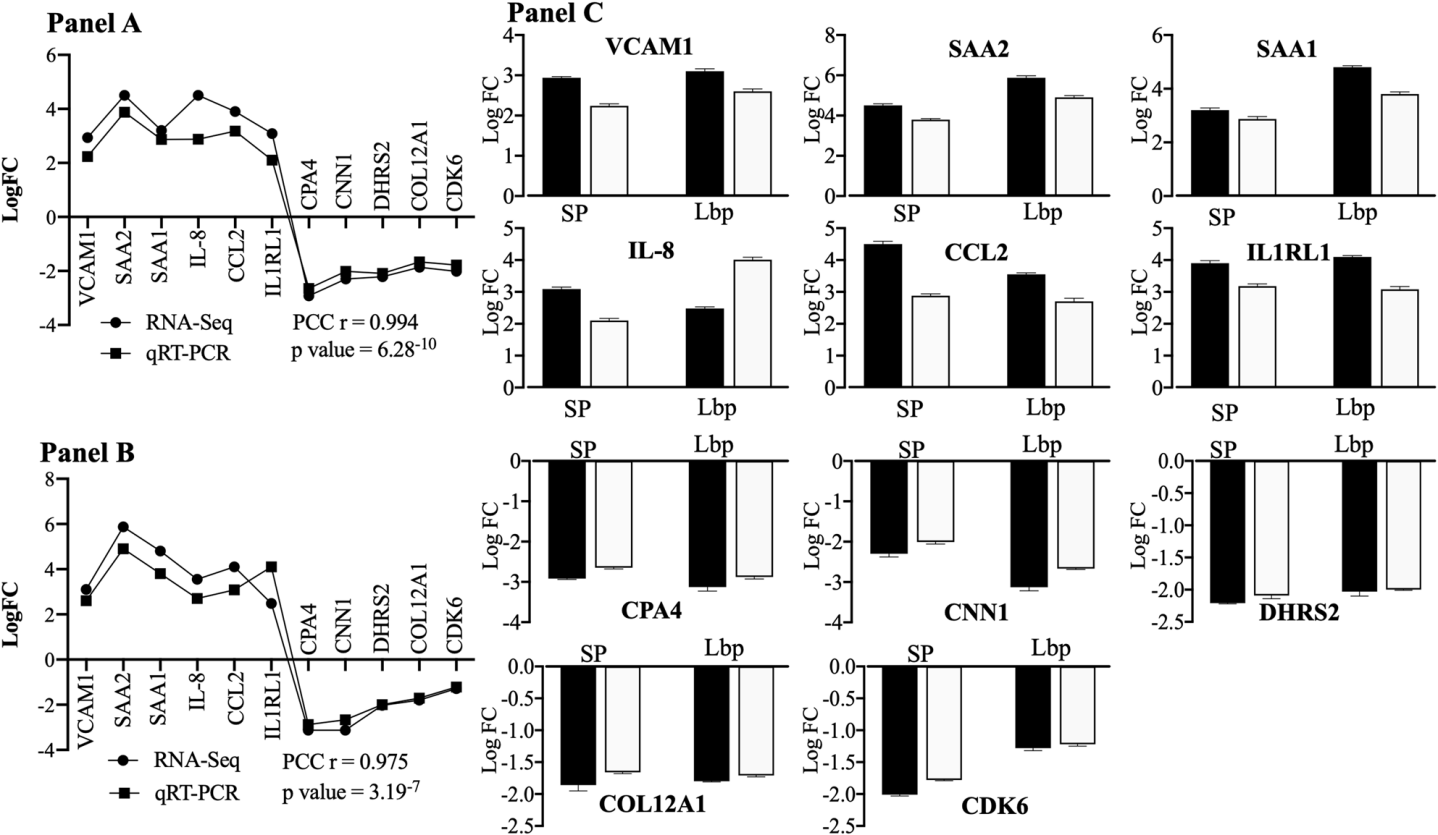
Validation of DEGs with qRT-PCR. (Panel **A**) Correlation of the gene expression (LogFC) of DEGs obtained from RNA-seq and qRT-PCR after incubation of hBMECs with *S. pneumoniae.* (Panel **B**) Correlation of the gene expression (LogFC) of DEGs obtained from RNA-seq and qRT-PCR after incubation of hBMECs with Lbp. (Panel ** C**). Gene expression (LogFC) of DEGs used to validate RNA-seq analysis of induced hBMECs (*S. pneumoniae* or Lbp). Black bars—LogFC values from RNA-seq, white bars—LogFC calculated from qRT-PCR.

To validate the results obtained from the RNA-seq analysis, a subset of 11 representative DEGs was analyzed with qRT-PCR (Table 1). Results from qRT-PCR were consistent to those obtained from RNA-seq with a high correlation, which was evaluated by the Pearson Correlation Coefficient (r = 0.994, p < 0.05; for hBMECs exposed to *S. pneumoniae* and r = 0.975, p < 0.05; for hBMECs induced with Lbp, Fig. 2). Thus, confirming reliability of data derived from RNA-seq analysis.

**Table 1:** Primers used in the study.

No	Protein/(Gene)	Primer	sequence (5′–3′)	Primer efficiency	Amplicon length (bp)	Annealing temperature (°C)
**Primers designed to perform qRT-PCR for gene validation**						
1	C–C motif chemokine ligand 2 (*CCL2*)	*CCL2*- sense	CAATCAATGCCCCAGTCACCT	94.99%	175	55
		*CCL2*- antisense	TCCTGAACCCACTTCTGCTTG			
2	Vascular cell adhesion molecule 1 (*VCAM1*)	*VCAM1*- sense	CCCTGAGCCCTGTGAGTTTT	98.01%	138	60
		*VCAM1*- antisense	GGCCACCACTCATCTCGATT			
3	Interleukin 8, C-X-C motif chemokine ligand 8 (*CXCL8*)	*CXCL8*- sense	CTCCAAACCTTTCCACCCCA	94.99%	153	55
		*CXCL8*- antisense	TTCTCCACAACCCTCTGCAC			
4	Serum amyloid A2 (*SAA2*)	*SAA2*- sense	CCTTGGTCCTGAGTGTCAGC	96.66%	150	60
		*SAA2*- antisense	CATAGTTCCCCCGAGCATGG			
5	Serum amyloid A1 (*SAA1*)	*SAA1*- sense	TTTTGATGGGGCTCGGGAC	95.02%	150	60
		*SAA1*- antisense	TCGGTGATCACTTCTGCAGC			
6	Interleukin 1 receptor like 1 (*IL1RL1*)	*IL1RL1*- sense	AAGGTACAGGGCGCACAAG	93.29%	150	60
		*IL1RL1*- antisense	CCTTGCTCATCCTTGACCGT			
7	Cyclin-dependent kinase 6	CDK6- sense	CCGTGGATCTCTGGAGTGTTG	98.10%	117	60
		CDK6- antisense	GGAGTCCAATCACGTCCAAGA			
8	Collagen alpha-1	COL12A1- sense	GACCAGCACTTCCCTCAAAGA	95.22%	114	60
		COL12A1- antisense	GGTCGGGTAGTTTCTTGAGCA			
9	Dehydrogenase/reductase SDR family member 2	DHRS2- sense	GCTGTCATCCTGGTCTCTTCC	96.54%	113	60
		DHRS2- antisense	AGCTCCAATGCCAGTGTTCTA			
10	Calponin-1	CNN1- sense	TGCGAATTCATCAATAAGCTGCA	97.12%	112	60
		CNN1- antisense	ACTTGGTGATGGCCTTGATGA			
11	Carboxypeptidase A4	CPA4- sense	ATTATGGAGGAAGACGCGGTC	97.67%	123	60
		CPA4- antisense	TACACTTCGGAGCAAGGGTTG			
12	β-microglobulin (house-keeping gene) (*b2m*)	*b2m*- sense	GCTCGCGCTACTCTCTCTTT	98.84%	134	55
		*b2m*- antisense	CGGATGGATGAAACCCAGACA			
**Primers designed to knock-out Lbp gene (SPΔLbp)**						
13	F1 fragment (351 bp upstream to Lbp gene)	*Ko Lbp-F1 F*	CCTTAGCCTACTTGATCCTGCT	-	351	60
		*Ko Lbp-F1 R*	TTCTACTCCTGAAAATTTAATCTGTCAAG			
14	Bla gene	*Ko Bla F*	CTTGACAGATTAAATTTTCAGGAGTAGAAATGAGTATTCAACATTTCCGTGTCG	-	913	60
		*Ko Bla R*	TGTCATTTTGTGGGTCTGACTCTTTACCAATGCTTAATCAGTGAGGC			
15	F2 fragment (300 bl downstream to Lbp)	*Ko Lbp-F2 F*	AGAGTCAGACCCACAAAATGACA	-	300	60
		*Ko Lbp-F2 R*	CAATTTGTTCGGCGTTGATCC

### Categorization of the DEGs according to GO-molecular function and GO-biological processes

Possible role of the Lbp in evoking cell-signaling events that may contribute to the onset of meningitis was investigated, for which GO-molecular function and GO-biological processes were taken into account. Biological processes induced by Lbp or *S. pneumoniae* were categorized using a peer-reviewed Reactome server (Tables 2 and 3; Supplementary dataset 1.6 and 1.7). Signaling pathways involving DEGs of both treatments and molecular functions were investigated using the peer-reviewed server –PaintOmics, which allow analysis of multiple omics-derived candidate datasets and automatically highlights the expression of genes participating in the statistically significant pathways identified by the server (Table 4; Supplementary dataset 1.8; Supplementary dataset 2.1 to 2.8, pathways presented in Supplementary datasets 2 are from KEGG)^33–35^. It is important to note that the pathways identifiers and pathway names assigned by each server may differ (Supplementary information Table S1).

**Table 2:** DEGs involved in the biological processes, categorized by Reactome server (transcriptomes induced after exposure of hBMECs to *S. pneumoniae*).

No	Pathway identifier	Pathway name	Entities found	Entities total	Entities FDR	Reactions found	Reactions total	Reactions ratio
1	R-HSA-1280215	Cytokine Signaling in Immune system	52	1055	4.62e−14	235	639	0.0533
2	R-HSA-6783783	Interleukin-10 signaling	13	86	4.62e−14	2	15	0.0013
3	R-HSA-449147	Signaling by Interleukins	36	640	2.08e−13	173	491	0.0410
4	R-HSA-6785807	Interleukin-4 and Interleukin-13 signaling	18	211	2.08e−13	15	46	0.0038
5	R-HSA-380108	Chemokine receptors bind chemokines	11	48	3.23e−05	7	18	0.0015
6	R-HSA-168256	Immune System	77	2663	0.004	414	1526	0.1273
7	R-HSA-909733	Interferon alpha/beta signaling	9	184	0.036	16	20	0.0017
8	R-HSA-380994	ATF4 activates genes	3	32	0.036	3	7	5.84e−04

**Table 3:** DEGs involved in the biological processes, categorized by Reactome server (transcriptomes induced after exposure of hBMECs to Lbp).

No	Pathway identifier	Pathway name	Number of entities found	Number of entities total	Number of entities FDR	Number of reactions found	Number of reactions total	Reactions ratio
1	R-HSA-1280215	Cytokine Signaling in Immune system	65	1055	8.90e−14	244	639	0.0533
2	R-HSA-6783783	Interleukin-10 signaling	13	86	1.34e−13	2	15	0.0013
3	R-HSA-449147	Signaling by Interleukins	40	640	5.45e−11	199	491	0.0410
4	R-HSA-6785807	Interleukin-4 and Interleukin-13 signaling	17	211	3.19e−09	25	46	0.0038
5	R-HSA-168256	Immune System	107	2663	2.06e−06	493	1526	0.1273
6	R-HSA-913531	Interferon signaling	21	392	2.06e−06	15	66	0.0055
7	R-HSA-380108	Chemokine receptors bind chemokines	13	48	3.59e−06	8	18	0.0015
8	R-HSA-909733	Interferon alpha/beta signaling	11	184	4.54e−04	4	20	0.0017
9	R-HSA-877300	Interferon gamma signaling	13	250	5.03e−04	4	15	0.0013
10	R-HSA-2559582	Senescence-Associated Secretory Phenotype (SASP)	8	89	0.009	15	22	0.0018
11	R-HSA-5676594	TNF receptor superfamily (TNFSF) members mediating non-canonical NF-kB pathway	5	17	0.027	9	12	0.0010
12	R-HSA-1474244	Extracellular matrix organization	25	329	0.027	93	318	0.0265
13	R-HSA-446107	Type I hemidesmosome assembly	4	11	0.044	6	6	5.01e−04
14	R-HSA-2022090	Assembly of collagen fibrils and other multimeric structures	9	67	0.046	10	26	0.0022

**Table 4:** Biological processes identified in the *S. pneumoniae*-induced and Lbp-induced transcriptomes of hBMECs by PaintOmics server.

No	ID	Pathway name	Unique genes	Gene expression	Combined pValue (Fisher)
1	hsa04668	TNF signaling pathway	32	1.24314E−05	1.24314E−05
2	hsa04145	Phagosome	6	0.004610938	0.004610938
3	hsa04062	Chemokine signaling pathway	18	0.006657922	0.006657922
4	hsa04514	Cell adhesion molecules (CAMs)	11	0.006793736	0.006793736
5	hsa04657	IL-17 signaling pathway	21	0.014268539	0.014268539
6	hsa04060	Cytokine-cytokine receptor interaction	41	0.014445153	0.014445153
7	hsa04064	NF-kappa B signaling pathway	18	0.023454686	0.023454686
8	hsa04151	PI3K-Akt signaling pathway	25	0.040049033	0.040049033
9	hsa05134	Legionellosis	11	0.000845211	0.000845211
10	hsa05133	Pertussis	14	0.020114002	0.020114002
11	hsa05152	Tuberculosis	16	0.022068474	0.022068474
12	hsa05150	*Staphylococcus aureus* infection	4	0.028018603	0.028018603

### Biological processes identified in the Lbp-induced hBMECs

Lbp is one of the promising surface proteins of *S. pneumoniae*, which was previously identified as a potential ligand of hBMECs in our laboratory^13^. In this study, we analyzed the gene expression of hBMECs exposed to this protein by RNA-seq. The functional analysis performed by Reactome server identified 14 pathways with FDR < 0.05 (Table 3). Seven out of those 14 pathways coincided with pathways identified in the independent analysis of *S. pneumoniae*-induced hBMECs transcriptome (Supplementary dataset 2.9) and several DEGs involved in those 7 pathways were common in both treatments (Supplementary dataset 1.9).

Below we have described the signaling pathways altered exclusively in the Lbp-induced transcriptome. Incubation of Lbp with hBMECs markedly evoked expression of genes related to biological pathways such as “Interferon signaling” (R-HSA-913531) and “Interferon-γ signaling” (R-HSA-877300), which accounted for 21 DEGs and 13 DEGs, respectively (Table 3). During the bacterial infection, interferon signaling plays a central role in mounting the immune response. We identified up-regulation of 19 DEGs participating in the interferon signaling. Among these genes, we observed slight up-regulation of genes encoding for guanylate-binding protein 1 to 5 (GBP1, LogFC 1.10; GBP2, LogFC 1.61; GBP3, LogFC 1.08; GBP4, LogFC 1.30 and GBP5, LogFC 2.78), which are described as cytosolic “glue trap” capturing cytosolic Gram-negative bacteria (Supplementary dataset 1.10). Moreover, Lbp induced expression of IFI35 (LogFC 1.31) and OAS1 (LogFC 1.31), which activate macrophages to release pro-inflamatory cytokines via NFκβ. Lbp also induced expression of a group of interferon-induced IFIT proteins (IFIT1, LogFC 1.09; IFIT2, LogFC 1.02 and IFIT3, LogFC 1.09). IFIT3 acts an inhibitor of cellular processes such as cell migration, proliferation exhibiting an antiproliferative activity.

On the other side, Lbp evoked genes participating in the “Extracellular matrix organization” pathway (R-HSA-1474244, 25 DEGs), which together with two pathways containing small number of DEGs (“Type I hemidesmosome assembly” -R-HSA-446107 and “Assembly of collagen fibrils and other multimeric structures” -R-HSA-2022090) were related to maintenance of cell and tissue structures and integrities. The microvessels in brain consist of specialized microvascular endothelial cells interconnected by tight junctions surrounded by extracellular matrix (ECM) components. Thus, down-regulation of genes encoding ECM-stabilizing proteins and compounds (COL12A1, LogFC − 1.80; plectin (PLEC, LogFC − 1.38; ITGB4, LogFC − 1.31 and LOXL1, LogFC − 1.24) and the Up-regulation of genes encoding integrins, laminins and molecules participating in cell-to-cell adhesion (CTSS, LogFC 3.56; VCAM1, LogFC 3.12; LAMB3, LogFC 2.40; CD47, LogFC 1.41; MMP1, LogFC 1.78) suggested an active remodeling of the ECM, which in turns facilitates entry of pathogens to the neurovascular unit (Supplementary dataset 1.10).

Several pathological conditions such as exposure to pathogens induce cellular senescence. In this study, Lbp evoked expression of 8 genes involved in the signaling pathway “Senescence-Associated Secretory Phenotype” (R-HSA-2559582). Senescence is an irreversible proliferation arrest that was induced by Lbp, which was evidenced by the down-regulation of CDK6 (LogFC − 1.29) participating in the control of cell cycle and proliferation and by the up-regulation of genes such as histone H2 (HIST1H2AC, LogFC 1.49; HIST2H2BE, LogFC 1.38 and HIST1H2BJ, LogFC 1.13), IL-1A (LogFC 1.49), IL-6 (LogFC 2.47), CXCL8 (LogFC 3.6), CEBPB (LogFC 1.10), which reinforce the cell cycle arrest (Supplementary dataset 1.10).

### Biological processes identified in the *S. pneumoniae*-induced hBMECs

*Streptococcus pneumoniae* has the ability to cross the BBB and generate strong inflammatory reactions that contributes substantially to the brain damage. Functional analysis of transcriptome revealed 8 pathways (statistical significance, FDR < 0.05) altered in hBMECs during infection (Table 2; Supplementary dataset 1.6). 5 out of 8 pathways were related to signaling mediated by cytokines such as IL-4, IL-13 and IL-10. 2 out of 8 pathways were related with cell immune response and one was identified as the pathway ATF4 (Table 2).

The biological pathways “Immune system” (R-HSA-168256), “Cytokine signaling in immune system” (R-HSA-1280215) and “Signaling by interleukins” (R-HSA-449147) accounted for the largest number of participating DEGs 77, 52 and 36, respectively (Table 2). These pathways play an important role in the immune response evoked by *S. pneumoniae* in hBMECs. Invasion of pneumococcus to hBMECs diminished expression of genes inducing the production of filamin-A (FLNA*,* LogFC − 1.30), filamin-B (FLNB, LogFC − 1.05), tubulin alpha 1 (TUBA1A, LogFC 1.12), fibronectin (FN1*,* LogFC − 1.05) and BST2 (LogFC − 1.02) (Supplementary dataset 1.11), which contribute in the internal formation of protein filaments that stabilize the cytoskeleton. Furthermore, invasion of *S. pneumoniae* to hBMECs evoked the over-regulation of genes implicated in cell activation, differentiation and proliferation contributing to *S. pneumoniae* clearance. *S. pneumoniae* is recognized by antigen-presenting cells via Toll-like receptors inducing the activation of factor nuclear kappa and leading the subsequent regulation of lymphocyte populations. Several DEGs implicated in these processes such as the HLA class I histocompatibility antigen (HLA-F, LogFG 1.03), nuclear factor kappa beta 2 (NFKB2, LogFG 1.32), nuclear factor kappa beta 1A (NFKBIA, LogFG 1.40), some interleukins such as IL-23A (LogFC 1.36), IL-1β (LogFC 1.42), IL-1α (LogFC 2.06), IL-6 (LogFC 2.47) as well as some interleukin receptors such as IL-7R (LogFC 1.15) and IL-18R1 (LogFC 2.59) were induced by pneumococci (Supplementary dataset 1.11). Additionally, we observed an up-regulation of NOD2 (LogFC 1.44), which is a member of the NOD-like receptors intracellular located that plays a role on innate immunity by detecting intracellular pathogen-associated patterns. We also observed a markedly overexpression of genes participating in the production of granulocytes such as granulocyte colony-stimulating factor (CSF3, LogFC 3.45). Simultaneously, over expression of the hypoxia-inducible factor 1-alpha (HIF1A, LogFC 1.03) indicated activation of active bacterial clearance and up-regulation of C3 (LogFC 2.3) and C1S (LogFC 1.23) showed activation of complement pathway (Supplementary dataset 1.11).

### Gene ontology of hBMECs transcriptomes induced by *S. pneumoniae* and Lbp

**Figure 3:**
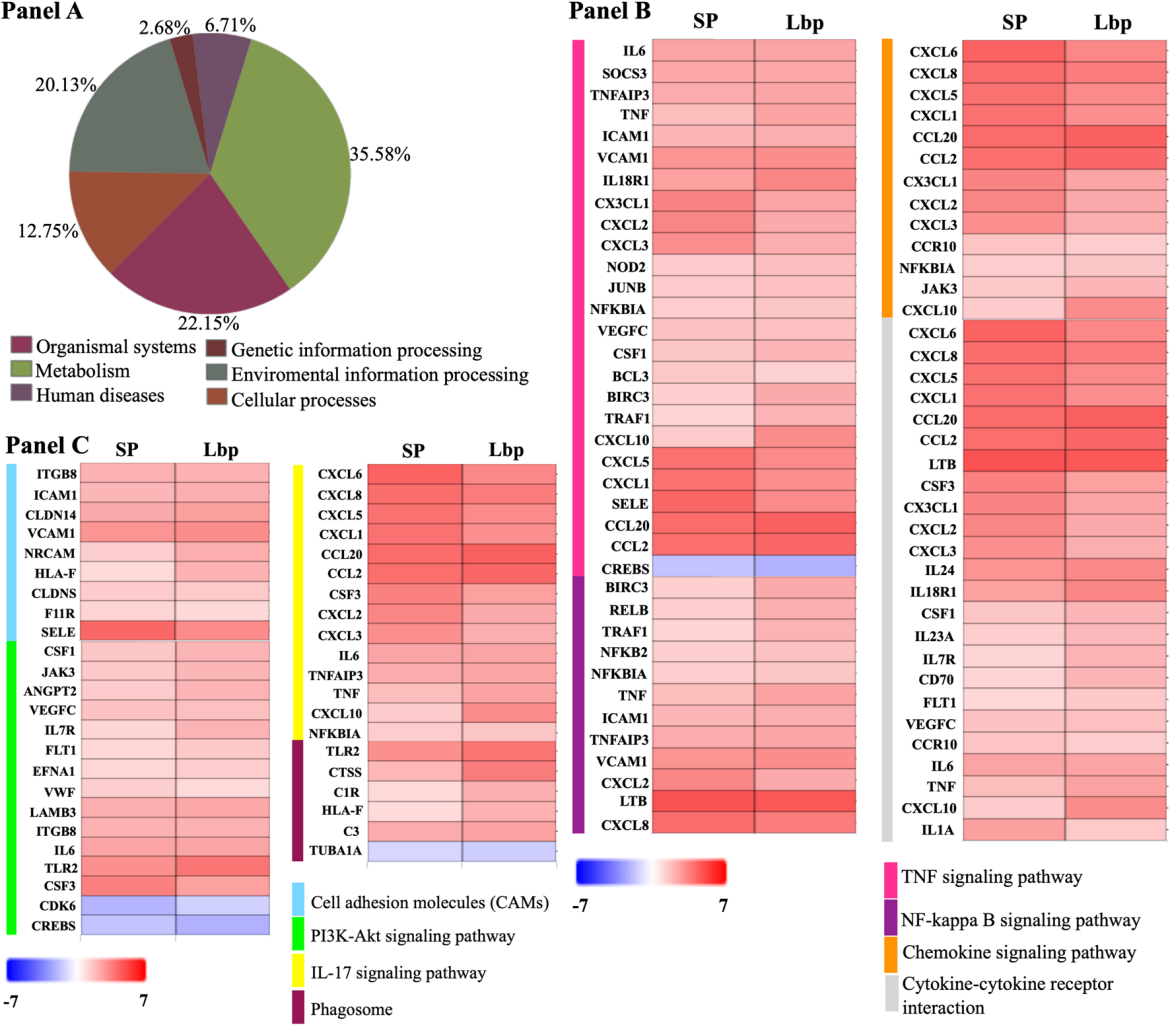
Functional analysis of DEGs identified in the *S. pneumoniae*-induced and Lbp-induced hBMECs transcriptome. (Panel **A**) represents Gene ontology molecular functions investigated with PaintOmics server. Pie chart representing functional categories automatically generated from DEGs induced in both treatments of hBMECs (*S. pneumoniae* or Lbp). (Panel **B**,**C**) show the integrative analysis of signaling pathways/GO biological processes. Heat maps showing statistically significant biological processes involved in the immune response identified by PaintOmics server. Red shaded genes—up-regulated, blue shaded genes—down-regulated. Shading intensity indicates the expression degree that each gene experienced. White color indicates no significant change in the gene expression. Range of the fold change (LogFC values) are presented in the scale. Color bars indicate biological processes. TNF signaling pathway, NF-kappa B signaling pathway, Chemokine signaling pathway and Cytokine-cytokine receptor interaction activated by *S. pneumoniae* (SP) and Lbp in hBMECs are displayed in (**B**). Cell adhesion molecules, PI3K-Akt signaling pathway, IL-17 signaling pathway, Phagosome) activated by *S. pneumoniae* (SP) and Lbp in hBMECs are shown in (Panel **C**).

Functional analysis was investigated on the DEGs of both treatments (*S. pneumoniae* and Lbp) using PaintOmics server. Analysis revealed that 12.75% DEGs participated in cellular processes, 20.13% DEGs were involved in environmental information processing, 2.68% DEGs were related with genomic information processing, 6.71% DEGs were associated with human diseases, 35.58% DEGs play a role in metabolism and 22.15% DEGs were part of the organismal system (Fig. 3A).

Biological processes (signaling pathways) activated in hBMECs after infection with *S. pneumoniae* and induced with Lbp were investigated. Despite seven signaling pathways were identified as common pathways in both transcriptomes (by Reactome), we could not assure that those pathways involving DEGs were statistically significant. To this regard, functional analysis seeking pathways altered by *S. pneumoniae* and Lbp was performed using PaintOmics. Functional analysis of biological processes revealed 12 statistically significant signaling pathways (Fisher combined p-value, p < 0.05) (Table 4). 8 out of 12 pathways were involved in the host immune response against *S. pneumoniae* (Table 4; Fig. 3B,C; Supplementary dataset 2.1 to 2.8) and 4 out of 12 were related to infectious processes caused by other pathogenic bacteria (Table 4; Supplementary dataset 2.10).

**Figure 4:**
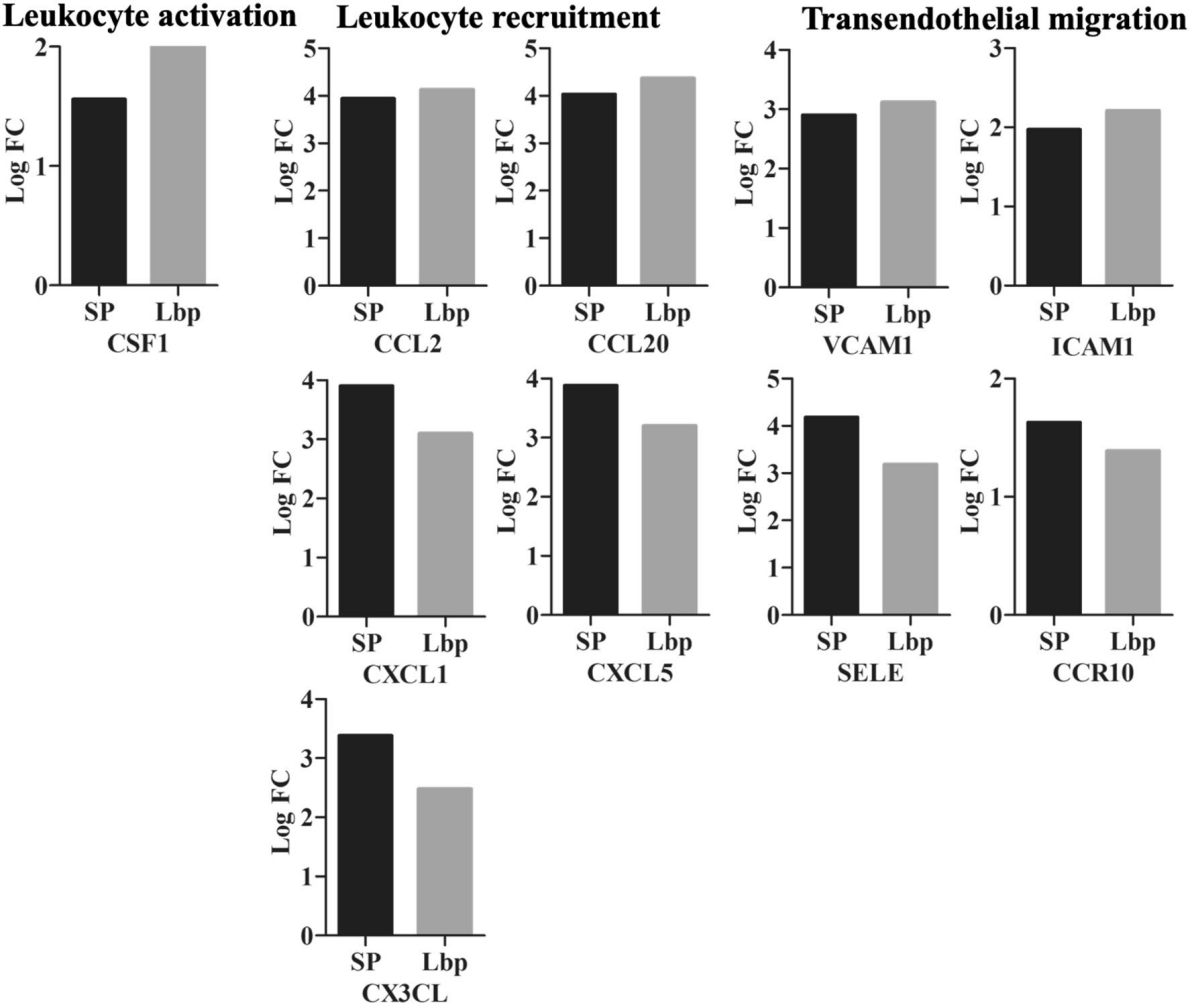
DEGs involved in the leucocyte recruitment, activation and transendothelial migration. Gene expression (LogFC values) of differentially expressed genes belonging to CXCL, CCL and TNF families participating in the leucocyte recruitment, activation and transendothelial migration are represented as bars. Black bars indicate DEGs identified in the *S. pneumoniae*-induced transcriptome and gray bars indicate DEGs.

Attachment of *S. pneumoniae* to the BBB is facilitated by cell adhesion molecules. In this study, we observed over expression of PECAM-1 (SP, LogFC 1.03), ICAM-1 (SP, LogFC 1.98; Lbp, LogFC 2.22) and VCAM-1 (SP, LogFC 2.90; Lbp, LogFC 3.13) (Fig. 3C; Supplementary dataset 2.1). Once pneumococcus enters the CNS, it continues multiplying and releasing highly immunogenic components that are recognized by Toll-like receptor 2, which induces innate immune response. In this study, up-regulation of TLR2 in both transcriptomes (SP, LogFC 3.04; Lbp, LogFC 3.79) negatively influenced the expression of CDK6 (SP, LogFC − 2.02; Lbp, LogFC − 1.28), which is important to control transition of G1 to S phase in the cell cycle (Supplementary dataset 2.2). As mentioned above (*S. pneumoniae*-induced transcriptome), NOD-like receptors (NLRs) recognize intracellular pathogen-associated patterns to induce innate immunity. We observed that both treatments evoked up-regulation of NOD2 (SP, LogFC 1.44; Lbp, LogFC 1.79) whose activation led to induction of nuclear factor kappa B (NF-κB) pathway for the subsequent up-regulation of pro-inflammatory mediators (Supplementary dataset 2.3). Over expression of members of the CXCL, CCL and TNF families such as CXCL1, CXCL2, CXCL3, CXCL5, CXCL8, CCL2, CCL20, CSF3 and IL6 was observed in both transcriptomes evidencing the leukocyte recruitment, leukocyte activation, transendothelial migration of leukocytes to the injured tissue and their subsequent degranulation (Fig. 4; Supplementary dataset 2.4 to 2.6). Moreover, these chemokines participate in the IL-17 signaling pathway, which is important for the protection of host against extracellular pathogens (Supplementary dataset 2.7). Additionally, over expression of HLA-F (SP, LogFC 1.02; Lbp, LogFC 2.16), CTSS (SP, LogFC 1.94; Lbp, LogFC 3.56), C1R (SP, LogFC 1.04; Lbp, LogFC 2.24) and C3 (SP, LogFC 2.30; Lbp, LogFC 2.49) evidenced the phagosome formation during active phagocytosis (Supplementary dataset 2.8).

### Deletion of Lbp gene altered gene expression in hBMECs in contrast to wild type *S. pneumoniae*

To corroborate the role of Lbp in evoking the cell response we challenged the cells with Lbp deletion mutant (SPΔLbp) and the expression of selected genes was compared with the expression levels induced by wild type *S. pneumoniae*. Similarly, to rule out that the cell response to Lbp challenge, observed in this study, was not merely because of the exogenous protein originated from *E.coli,* we challenged the cells with enhanced green fluorescent protein (eGFP overexpressed in *E.coli*, a benign protein control) and the cell response was compared with the gene expression induced by Lbp.

A DNA cassette (5′-351 bp upstream of *Lbp—bla* gene encoding beta lactamase—300 bp downstream to *Lbp* gene-3′) constructed to delete *Lbp* by homologous recombination is presented in Supplementary information Fig. S11. *S. pneumoniae* was transformed with the cassette and transformants were selected in the presence of carbenicillin. Replacement of *Lbp* by *bla* in SPΔLbp confirmed with sequencing is presented in Supplementary information Fig. S11.

**Figure 5:**
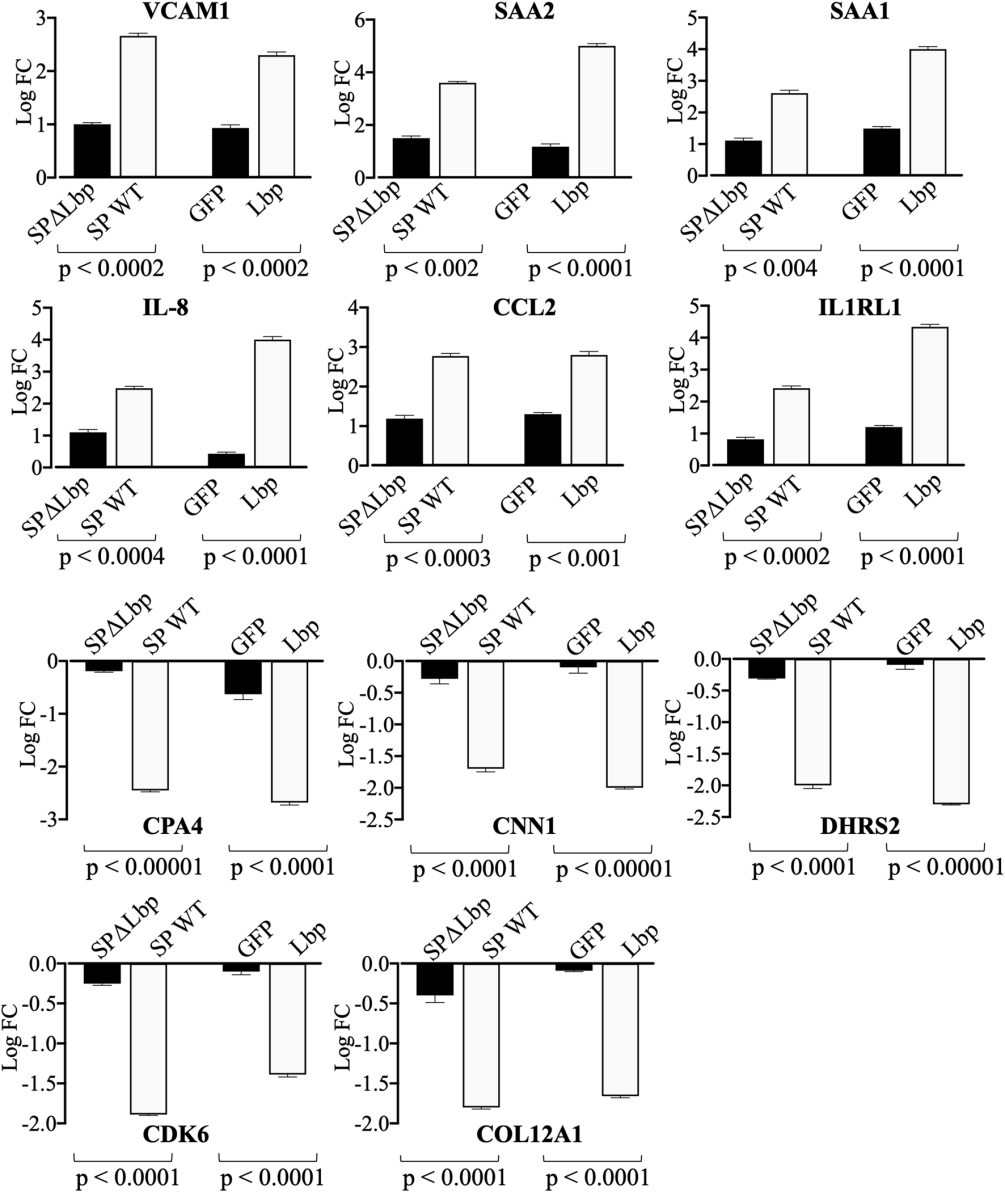
Comparison of expression of selected genes in hBMECs. Comparison was performed between hBMECs induced by *S. pneumoniae* wild type (SP WT) and *S. pneumoniae* Δ *Lbp* (SPΔLbp) using qRT-PCR. Comparison was also performed between hBMECs induced with GFP and Lbp using same set of the genes. Statistically significant difference (p < 0.01, two-tailed p value) was calculated by unpaired *t* test. Sta*t*istics was performed with on-line statistics tool of GraphPad https://www.graphpad.com/quickcalcs/ttest1.

Expression of VCAM-1, SAA2, SAA1, IL-8, CCL2 and IL1RL1 in hBMECs was significantly decreased (p < 0.01, unpaired two-tailed *t* test, Fig. 5) in SPΔLbp compared to wild type *S. pneumoniae*. While, downregulation of CPA4, CNN1, DHRS2, CDK6 and COL12A1 observed in hBMECs induced with wild type *S. pneumoniae* was abolished in case of SPΔLbp (Fig. 5). These results indicate that Lbp plays an important role in inducing the signalling events in hBMECs. It is noteworthy that, deletion of *Lbp* did not abolish the differential expression of the genes (mainly upregulated), however significant reduction in the expression was observed. This result may indicate a minor contribution of other adhesins (e.g. CbpA, RrgA, Nan, etc.) in the induction of cell signaling in comparison with Lbp, which exerts a remarkable impact.

## Discussion

*Streptococcus pneumoniae* invades the neurovascular unit by crossing the BBB via transcellular route or by disrupting the tight junctions, which enhances the barrier instability^36,37^. Transcellular translocation of *S. pneumoniae* is facilitated by interaction of surface bacterial compounds with the host’s cell receptors. Hitherto, several surface proteins such as CbpA, RrgA, Nan and Ply have shown to promote pneumococcal invasion of brain endothelial cells^38–41^. Recently, we also have identified pneumococcal protein ligands that interact with hBMECs, among them Lbp showed a strong interaction with endothelial cells^13^. Similarly to our previous results indicating the surface exposure of pneumococcal Lbp protein, Lbp of *S. pyogenes* has been described as a membrane protein and the interaction with laminin was shown to be mediated via zinc bonds^13,42^. Thus, in this study using RNA-seq technology we attempted to decipher the cell signaling events triggered in hBMECs upon adhesion of Lbp. In addition, an integrative functional analysis (DEGs induced by *S. pneumoniae* and Lbp) was executed for a better understanding of *S. pneumoniae* invasion across BBB. To confirm that the effect observed was caused by Lbp and not by contaminants originated from *E.coli*, we evaluated the cell response against eGFP that was produced using the same *E.coli* expression system. Although we observed a small alteration in the gene expression in eGFP challenged cells, the cell response caused by Lbp protein was significantly higher (Fig. 5). In addition, the significant reduction in cell response to SPΔLbp compared with wild type *S. pneumoniae* indicates that Lbp is one of the major surface proteins that evokes signaling events in endothelial cells of brain microvasculature.

In this study, genes evoked in both treatments (*S. pneumoniae* and Lbp) showed same trend of expression, thus it is tempting to speculate that this consistency might be due to the similarity of signaling events induced by *S. pneumoniae* and Lbp. The common signaling pathways observed in both treatments as well as pathways specifically induced either by *S. pneumoniae* or Lbp provided us insight into the plausible role of adhesion lipoprotein. The molecular events evoked in both conditions are discussed below. To facilitate the comprehension of the gene expression, LogFC value is referred in parenthesis for some genes.

Entry of pneumococcus to the brain is facilitated by adhesins expressed on the cell surface^18,19^. We found up-regulation of ICAM-1, VCAM-1 and NRCAM in both transcriptomes (Supplementary dataset 2.1) and slight up-regulation of PECAM-1 (SP 1.02) in the *S. pneumoniae*-induced cells. PECAM-1 has been previously described to interact with the lectin-like domain of the pneumococcal neuraminidase A (NanA) contributing to the attachment of bacteria to the BBB^39,43^. In the receptor-mediated translocation, choline-binding protein (CbpA) recognizes the pIgR molecule facilitating entry of *S. pneumoniae* to the neurovascular unit^44^. We observed induced expression of several cell surface receptors in both transcriptomes (e.g. EFNA-1: SP 1.12, Lbp 1.37; FLT-1: SP 1.09, Lbp 1.51) participating in the PI3K-AKT signaling pathway, which is activated after cellular stimuli.

It is well known that pneumococcus experiences autolysis releasing bacterial components to the extracellular milieu, which in turn activates and enhances host immune response^45^. We found that several signaling pathways (*e.g*. Cytokine Signaling in Immune system, Signaling by Interleukins, Chemokine receptors bind chemokines, Interferon alpha/beta signaling, etc.) involved in cell immune response were induced independently in both hBMECs transcriptomes. Moreover, the integrative analysis showed participation of DEGs in common pathways (e.g. Cytokine-cytokine receptor interaction, Chemokine signaling pathway, TNF signaling pathway, NF-kappa B signaling pathway, etc.). *S. pneumoniae* is recognized by antigen-presenting cells via pattern recognition receptors. Immune activation occurs through toll-like receptor-2 (TLR-2) by recognition of pathogen-associated molecular patterns^46^. From the integrative analysis, we observed that TLR-2 was evoked in both transcriptomes. TLR-2-dependent pathway is enough to cause inflammation by stimulating the production of pro-inflammatory cytokines and chemokines^46,47^. In this study, both treatments induced the over expression of several cytokines such as IL-24, IL-1A and IL-23A and some chemokines (*e.g.* CXCL1, CXCL2, CXCL3, CXCL5, CXCL6) to activate white blood cells and to promote leukocyte chemotaxis for inducing pleocytosis. Early immune response is associated with activation of TNF-α, IL-6 and IL-1β^11,48,49^, which in turns is associated with an increased permeability of the BBB caused either by bacterial components released during pneumococcal autolysis (*e.g.* peptidoglycan, lipoteichoic acid) or by matrix metalloproteinases degrading ECM^3,50^. We observed over expression of TNF-α in both treatments (SP 1.82, Lbp 2.49), IL-6 (SP 2.48, Lbp 1.49) and up-regulation of MMP-1 (Lbp 1.78) only in the Lbp-induced transcriptome. The integrative analysis also showed induction of IL-17 signaling pathway. In this pathway IL-17 family plays role in acute and chronic inflammatory responses protecting the host against extracellular pathogens^51,52^. DEGs evoked in this study from the CXC and CC subfamily were identified as participants of this pathway (CXCL8: SP 3.99, Lbp 3.60; CCL2: SP 3.95, Lbp 4.14; CCL20: SP 4.03, Lbp 4.37; TNF: SP 1.82, Lbp 2.49 and CSF3: SP 3.45, Lbp 2.60). Additionally, from the integrative analysis we observed the activation of TNF signaling pathways by both *S. pneumoniae* and Lbp. DEGs participating in this route (CXC subfamily above mentioned) trigger the leukocyte activation and recruitment, participate as cell adhesion molecules (ICAM-1, VCAM-1 and SELE) and involve in the intracellular negative signaling (BCL3, SOCS3 and TNFAIP3) regulating the cytokine signaling. Additionally, NOD-like receptor 2 (playing a role in this pathway) was evoked in both treatments. NOD-like receptors are the molecules which detect pathogen-associated molecular patterns^53^. Specifically, NOD-2 has been described to sense muramyl peptides from pneumococcal peptidoglycan to enhance innate immune response^54^. Moreover, NOD-2 is described to activate a NF-κβ-dependent pro-inflammatory gene expression^55^. In this study, the NF-κβ signaling pathway was activated involving up-regulation of BIRC3 for activation of NFKBI and further stimulation of TRAF1 and NFKB2 to induce and regulate the signal transduction during inflammation. Activation of NOD2 is also described to occur after delivery of pneumococcal peptidoglycan fragments produced during the lysozyme-dependent digestion of *S. pneumoniae* in macrophages (phagocytosis)^56^. Additionally, we identified an increased expression of integrins (*e.g.* ICAM-1) facilitating the adhesion of macrophages to the microvascular epithelium to contribute with the pneumococcal clearance. The integrative analysis, performed with the total of DEGs identified in the *S. pneumoniae*-induced and Lbp-induced transcriptomes, revealed the activation of the phagosome pathway. Up-regulation of C1R, C1S and C3 indicate activity of the complement cascade and up-regulation of Cathepsin S (CTSS) indicates active degradation of antigenic proteins to peptides for presentation to the MHC class II for further lymphocyte stimulation. Overall, *S. pneumoniae* and Lbp activated biological processes that contribute to the bacterial clearance by classic innate and adaptive immune responses.

It is noteworthy that, Lbp evoked Interferon signaling by up-regulating expression of interferon-induced guanylate-binding proteins encoding by *GBP1*-*5*, which have been described to act as a cytosolic “glue trap” capturing the bacteria^57^. Additionally, Lbp activated the non-canonical NF-κβ signaling pathway for enhancing the lymphocyte adhesion to the microvascular epithelium via ICAM-1. It has been reported that some pathogens activate this pathway^58^. Furthermore, we observed that Lbp evoked biological processes involved in the cell structure organization such as the extracellular matrix organization, Type-I hemidesmosome assembly, and assembly of collagen fibrils and other multimeric structures. Although, these three processes were not statistically significant in *S. pneumoniae* treatment, several genes participating in cell structure maintenance such as FLNA, FLNB, BST2 were down regulated. In the following lines, these three processes are discussed together for practical purposes.

Extracellular matrix (ECM) is formed by structural components providing stability to endothelial cells and is involved in tissue homeostasis preventing bacterial invasion^59^. We identified over expression of genes stimulating production of laminins (LAMB3) and collagen (COL7A1, COL25A1). Additionally, DEGs encoding proteins regulating tight junction assembly (CD321), stimulating collagen production (ADAMTS3) as well as promoting adhesion to the endothelial cells (FBLN5) were up-regulated suggesting an active remodeling of ECM. Proteolytic cleavage of the ECM is required for the bacterial invasion into host tissues. Pathogenic bacteria recruit host proteases or inhibit expression of genes encoding structural compounds^60^. Lbp caused down-regulation of genes encoding collagen type XII (COL12A1 − 1.80) and integrins (ITGB4 − 1.31) as well as essential genes for the biogenesis of connective tissue (LOXL1). Additionally, Lbp evoked expression of the collagenase MMP1 (1.78), which is involved in the breakdown of ECM, which in turn may contribute to invasion of *S. pneumoniae* to the neurovascular unit. Furthermore, Lbp had effect on the type I hemidesmosome assembly and the assembly of collagen fibrils pathways. Type I hemidesmosomes are specialized junctional complexes connecting cytoskeleton of the cells to the ECM, while assembly of collagen fibrils refers the molecular mechanisms involved in the formation of higher collagen structures within the extracellular space^61,62^. Thus, these pathways play a role in the maintenance of tissue structure and integrity preserving the mechanical and physical properties of tissues. Lbp evoked expression of genes encoding laminin and laminin extracellular glycoproteins (LAMB3 and LAMC2) and caused down-regulation of gene encoding integrin (ITGB4) and plectin (PLEC), which interacts with molecules from the cell cytoskeleton providing support and strength to the cell. Overall, these findings suggest increased ECM instability facilitating permeability of BBB favoring the streptococcal invasion.

Laminin-binding proteins of *Streptococcus* share high homology to AdcAII*.* AdcAII is a zinc-binding lipoprotein that is important for infectivity of *S. pneumoniae*. Expression of AdcAII depends on the concentration of Zn^2+^. Under lower concentrations of Zn^2+^ the growth and colonization of *S. pneumoniae* seems to be affected. Interaction between laminin and AdcAII has been investigated previously by Brown et al. but no evidence of such interaction was found using dot blot experimets^63^. Previously, we have reported strong affinity between laminin binding protein (Spr0906) and BMECs^13^. In this study we have investigated the biological process altered in BMECs by Lbp but no evidence in the Zn-uptake was identified.

Finally, we identified induction of cell senescence by treatment of hBMECs with Lbp. Senescence is an irreversible proliferation arrest in which cell experiences changes in function, morphology and gene expression^64^. Licastro and Porcellini have suggested a close relation between CNS infections and senescence^65^. Lbp induced over expression of genes such as HIST1H2AC (responsible for DNA packaging) and CEBPB (stimulating transcription of CDKN2B), which reinforce the cell cycle arrest and down-regulation of CDK6 (cell division protein kinase 6, which is essential for cell cycle progression), respectively.

## Conclusion

*Streptococcus pneumoniae* is an extracellular pathogen capable to invade the central nervous system and cause meningitis. Surface proteins of *S. pneumoniae* play a critical role in neuroinvasion. We recently reported interaction between Lbp with hBMECs. In this study, we comprehensive picture of the hBMECs response against Lbp as well as intact bacteria by using a high-throughput RNA-seq technology. Both Lbp and *S. pneumoniae* seem to induce host immune response with activation of cytokines and chemokines. Based on the signaling pathways identified from the bioinformatics analysis, Lbp seem to induce signaling pathways involved in the breakdown and remodeling of ECM as well as cell senescence. This investigation revealed overall cell signaling events occurred in the microvascular endothelium upon adhesion *S. pneumoniae* to the cell surface. Furthermore, it provides plausible importance of the Lbp adhesin of *S. pneumoniae* in evoking the signaling events in the endothelium of the brain microvascular, which may promote pneumococcal invasion to the brain.

## Materials and methods

### Culture of human brain microvascular endothelial cells

hBMECs (D3 cell line), were obtained from Merck/Millipore (Prague, Czech Republic). Details are in the supplementary information Method S1. Cells were either harvested for RNA isolation (non-induced cell control) or incubated with live *S. pneumoniae* or Lbp.

### Bacterial strain

The neuroinvasive strain of *S. pneumoniae* (clinical isolate SPH) used in this study was isolated from the cerebrospinal fluid of a meningitis-suffering patient hospitalized in Louis Pasteur Hospital, Kosice, Slovakia. Cerebrospinal fluid was received in microbiological diagnostic laboratory to identify the causative agent of the meningitis. Use of the isolate SPH for experimental purpose was in accordance with the guidelines and regulations set by the ethical committee of the Hospital and diagnostic laboratory of the hospital. The isolate SPH was characterized by phenotyping (biochemical tests) and genotyping (sequencing of *lytA* and *rpoB* genes) in the hospital laboratory. *S. pneumoniae* was plated on Columbia agar blood base containing 5% (v/v) sheep blood and single isolated colony was grown as described in our previous publication^13^. *S. pneumoniae* were washed with minimal essential medium (Sigma) prior incubation with hBMECs. Number of the bacteria per microliter was measured with flow-cytometry.

### Synthesis of recombinant Lbp

Lbp was overexpressed in *E.coli* as described in our previous publication^13^. In short, the gene fragment encoding Lbp was amplified by PCR from genomic DNA of *S. pneumoniae*. Sense and antisense primer sequences, overhangs of restriction sites used for downstream cloning and amplicon length are shown in supplementary information Table S2. Amplified fragments were digested and ligated into pQE-30-mCherry plasmid (in-house modified vector pQE-30 UA, Qiagen, Supplementary information Fig. S1). Details of the digestion of amplified PCR product, ligation into a pQE-30-mCherry-STOP plasmid, transformation and selection of clones are presented in supplementary information Method S2. Presence of encoding gene was confirmed by sequencing (vector specific primers UA Insertom F and R, presented in Supplementary information Table S3).

Protein expression and purification were performed as described earlier^13^ (details are in supplementary information Methods S2 and S3). Purity of recombinant Lbp was evaluated by SDS-PAGE and MALDI-TOF MS (details are presented in supplementary information Method S4). Protein concentration was measured by Bradford method and aliquots of purified proteins were stored at − 20 °C in 20% glycerol until use.

### Challenge of hBMECs

Monolayer of hBMECs was cultured on 6-well plates and incubated either with live *S. pneumoniae* cells (MOI 0.5/well) or with recombinant Lbp (approximately 1 nMol, 27 μg/well) or with culture medium (non-induced control) for 6 h at 37 °C under 5% CO_2_ atmosphere. After incubation, culture media was removed, hBMECs were washed and cells were scrapped for RNA isolation.

### RNA isolation from hBMECs

mRNA from hBMECs was isolated using RNeasy Mini Kit (Qiagen, Germany) according to manufacturer’s instructions. During RNA isolation, samples were treated with DNAseI (Qiagen). RNA samples were eluted in RNase free water and quantified by nanodrop (Thermo Scientific). Integrity of RNA was monitored in 1% borax gel electrophoresis at 100 V and by capillary electrophoresis (Fragment analyzer, Advanced Analytical Technologies, Inc., USA). Samples were stored at − 80 °C until used for library preparation.

### Preparation of RNA libraries

RNA libraries were prepared exactly as described before in our publication^66^. In brief, 250 ng of RNA were reverse transcribed with oligodT primers for synthesis of the first strand cDNA using QuantSeq 3′ mRNA-Seq Library Prep Kit (Lexogen, Austria) as per manufacturer’s instructions. RNA template was removed and second strand was synthetized by using random hexamer containing Illumina-compatible linker sequences at its 5′ end. Double strand DNA library was purified using magnetic beads provided in the kit. Each library was amplified by PCR using unique single indexing i7 primers to add complete adapter sequence required for cluster generation and to generate sufficient DNA for sequencing and quality control. The number of cycles in PCR for each library was determined using PCR Add-on kit for Illumina (Lexogen). Number of cycles used for library amplification were as follows: hBMECs induced with *S. pneumoniae—*20 cycles, hBMECs induced with Lbp—20 cycles and non-induced cells—17 cycles. Amplified libraries were purified using magnetic beads supplied in the kit. Quality of the library and length of the fragments were checked on fragment analyzer.

### RNA sequencing

Libraries were sequenced on Illumina NextSeq, single-end 75 bp, to a minimal depth 8 million reads per sample. STAR aligner was used to process Fastq files, aligned to reference genome (*Homo sapiens* GRCh38) and generate gene counts. Differential gene expression analysis was carried out by R package edgeR. Data segregation to generate the final relation of differentially expressed genes (DEGs) between the challenged hBMECs (*S. pneumoniae* or Lbp) was performed using Excel (MS office). Differentially expressed genes (DEGs) included genes with a minimum average logCPM (count per million) of 2 and genes with logFC (fold change) ranging beyond ± 1. DEGs with *p*-value p > 0.01 were removed.

### Bioinformatic analysis of hBMECs transcriptomes

Raw RNA-seq data and processed data showing DEGs were deposited to EBI Arrayexpress repository (https://www.ebi.ac.uk/arrayexpress/) deposited under accession number E-MTAB-8054.

Venn diagram was created to display the relation between the hBMECs transcriptomes (*S. pneumoniae* or Lbp) (http://bioinfogp.cnb.csic.es/tools/venny/). Functional analysis of each set of DEGs (hBMECs challenged with *S. pneumoniae* or Lbp) was performed by Reactome server (https://reactome.org/). Common signaling pathways between hBMECs transcriptomes were investigated by PaintOmics (http://www.paintomics.org). DEGs involved in the statistically significant common pathways were automatically drawn by this web tool. Heatmapper server (http://www.heatmapper.ca/expression/) was used to group DEGs into GO biological processes and to construct heat maps.

### Validation of differentially expressed genes by qRT-PCR

RNA was reverse transcribed into cDNA using random hexamers (Thermo Scientific) exactly as described in our previous publication^66^. Briefly, 1 μg of RNA and 100 pMol of random hexamer were mixed and incubated 5 min at 65 °C (thermocycler Bio-Rad Laboratories, USA). Subsequently, 4 μL 5 × reaction buffer (Thermo Fisher Scientific, USA), 2 μL dNTP (10 mM), 1 μL RevertAid reverse transcriptase (200 U) (Thermo Fisher Scientific, USA) and 0.5 μL RiboLock RNase inhibitor (20 U) (Thermo Fisher Scientific, USA) were added. The reaction mixture was incubated 10 min at 25 °C, 1 h at 42 °C followed by 70 °C for 10 min.

A set of 11 DEGs significantly up and down regulated in RNAseq were selected for validation. Primers used in qRT-PCR (Table 1) were designed using Geneious Pro software (Biomatters, USA). Reaction mix of qRT-PCR was composed of 6 ng of cDNA, 1 × qPCR GreenMaster with highROX (Jena Bioscience, Germany), gene specific primers (10 pMol each) and RNase free water up to total volume 20 μL. Each DEG was evaluated in triplicates. Amplification cycle was as follows: 95 °C—10 min, 40 × [95 °C—15 s, 50–60 °C—30 s (annealing temperature varied according to the primers used), 72 °C for 30 s. (signal capture)], melting curve 60 °C to 95 °C—0.3% temperature increment/s (StepOnePlus, Thermo Fisher Scientific, USA). The gene expression (ΔΔCt) was normalized to β-2-microtubulin (house-keeping gene) as described before^67^. ΔΔCt values were converted to logFC (http://www.endmemo.com/algebra/log2.php). Correlation of expression values for DEGs obtained from RNA-seq and qRT-PCR was determined by calculating the Pearson correlation coefficient (r) using Graphpad Prism version 8.

### *Lbp* knock-out mutant (SPΔLbp)

*Lbp* gene was replaced with *bla* gene, encoding the beta-lactamase. Details of the cassette designed with overlapping extension PCR (OE-PCR) are presented in supplementary information Method S5 and supplementary information Fig. S11. Steps in the transformation of *S. pneumoniae* and selection of the Δ*Lbp* mutants are explained in supplementary information Method S6.

### Comparative analysis of gene expression in hBMECs: SPΔLbp vs. wild type S. pneumoniae and GFP vs. Lbp

hBMECs were incubated either with SPΔLbp or wild type (both MOI 0.5/well) or eGFP or Lbp (both 1 nMol/well) for 6 h. Non-induced cells were kept as a negative control. RNA was isolated from induced and non-induced cells, reverse transcribed and used in qRT-PCR as described above. Please note that, eGFP was overexpressed and purified in the same way as Lbp. The logFC was calculated as described above. Unpaired *t* test (https://www.graphpad.com/quickcalcs/ttest1) was used to assess statistical difference between logFC observed in wild type vs. SPΔLbp or GFP vs. Lbp. The experiment was performed in biological triplicates.

## Supplementary Information

Supplementary Information 1.

Supplementary Information 2.

Supplementary Information 3.

## Data Availability

The datasets generated and/or analyzed during the current study are available from the corresponding author on reasonable request.
