# Supplementary material for: Transcriptomic analysis of human brain microvascular endothelial cells exposed to laminin binding protein (adhesion lipoprotein) and Streptococcus pneumoniae
Source: Sci Rep. 2021 Apr 12;11:7970. doi: 10.1038/s41598-021-87021-4 (PMC8041795; doi:10.1038/s41598-021-87021-4)
Supplement: Supplementary file 2 — Supplementary Information 2. [file 41598_2021_87021_MOESM2_ESM.pdf]

Supplementary dataset 2.1. Schematic view of Cell adhesion molecules.
Molecules expressed in the cell surface leading to Leukocyte transendothelial migration or participating in the complement cascade. Expressed genes in the hBMECs challenged with *S. pneumoniae* and adhesion lipoprotein Lbp are highlighted according to the scale (range of the fold change, LogFC values). Red shaded boxes indicate up-regulated genes and blue shaded boxes indicate down-regulated genes. Each box was automatically divided to show the gene expression, left half displays gene expression induced by *S. pneumoniae* while right half displays the gene expression evoked by Lbp. Genes induced in hBMECs by both treatments (*S. pneumoniae* or Lbp) are indicated as fully colored boxes and are marked with a red star in the right upper corner. Pathways are taken from KEGG pathways (Kanehisa, M. and Goto, S.; KEGG: Kyoto Encyclopedia of Genes and Genomes. Nucleic Acids Res. 28, 27-30 (2000))

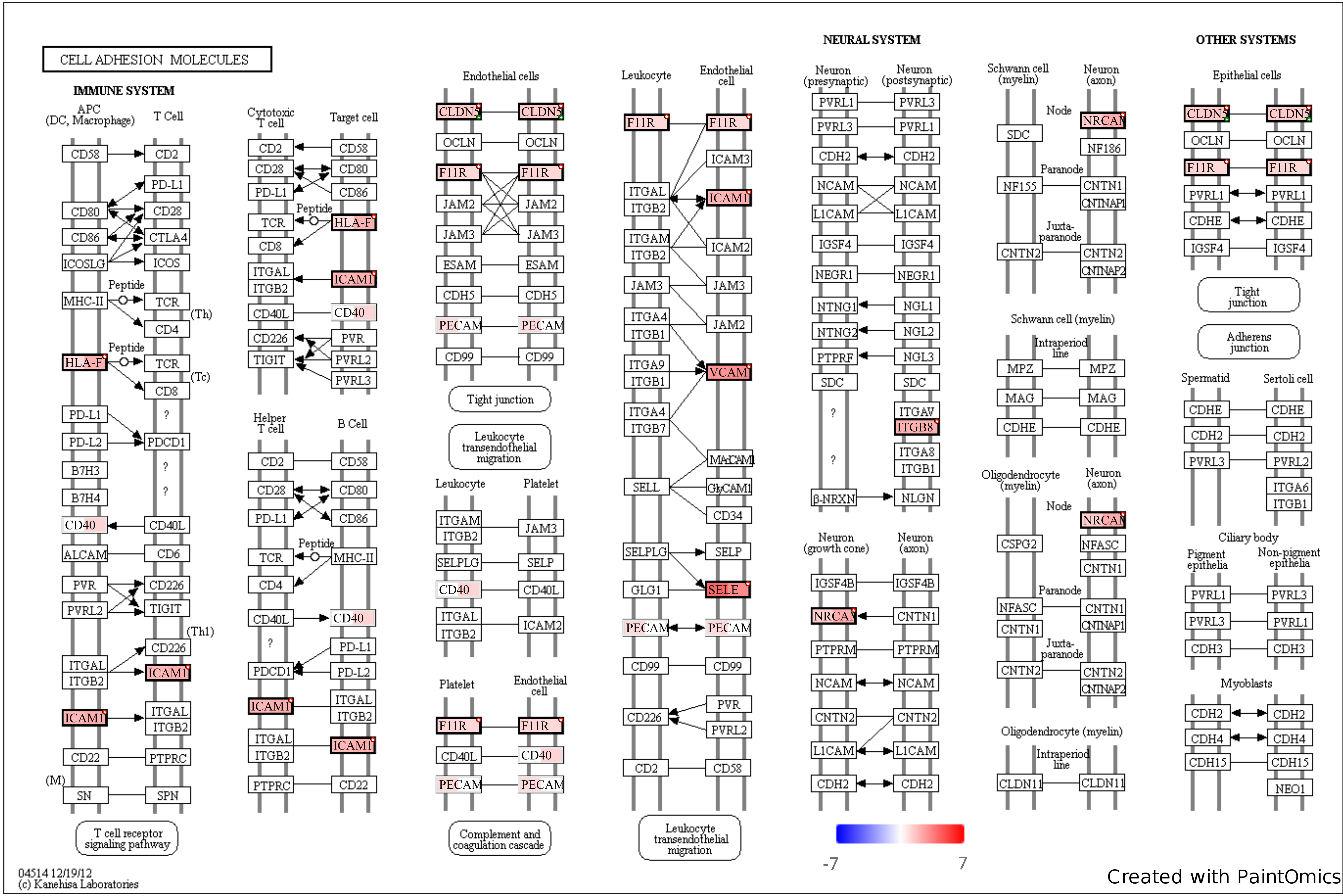

Supplementary dataset 2.2. Schematic view of PI3K-AKT signaling pathway. Pathway activated by many types of cellular stimuli or toxic insults. Events regulating cell cycle progression and key cellular processes. Expressed genes in the hBMECs challenged with *S. pneumoniae* and adhesion lipoprotein Lbp are highlighted according to the scale (range of the fold change, LogFC values). Red shaded boxes indicate up-regulated genes and blue shaded boxes indicate down-regulated genes. Each box was automatically divided to show the gene expression, left half displays gene expression induced by *S. pneumoniae* while right half displays the gene expression evoked by Lbp. Genes induced in hBMECs by both treatments (*S. pneumoniae* or Lbp) are indicated as fully colored boxes and are marked with a red star in the right upper corner.
Pathways are taken from KEGG pathways (Kanehisa, M. and Goto, S.; KEGG: Kyoto Encyclopedia of Genes and Genomes. Nucleic Acids Res. 28, 27-30 (2000))

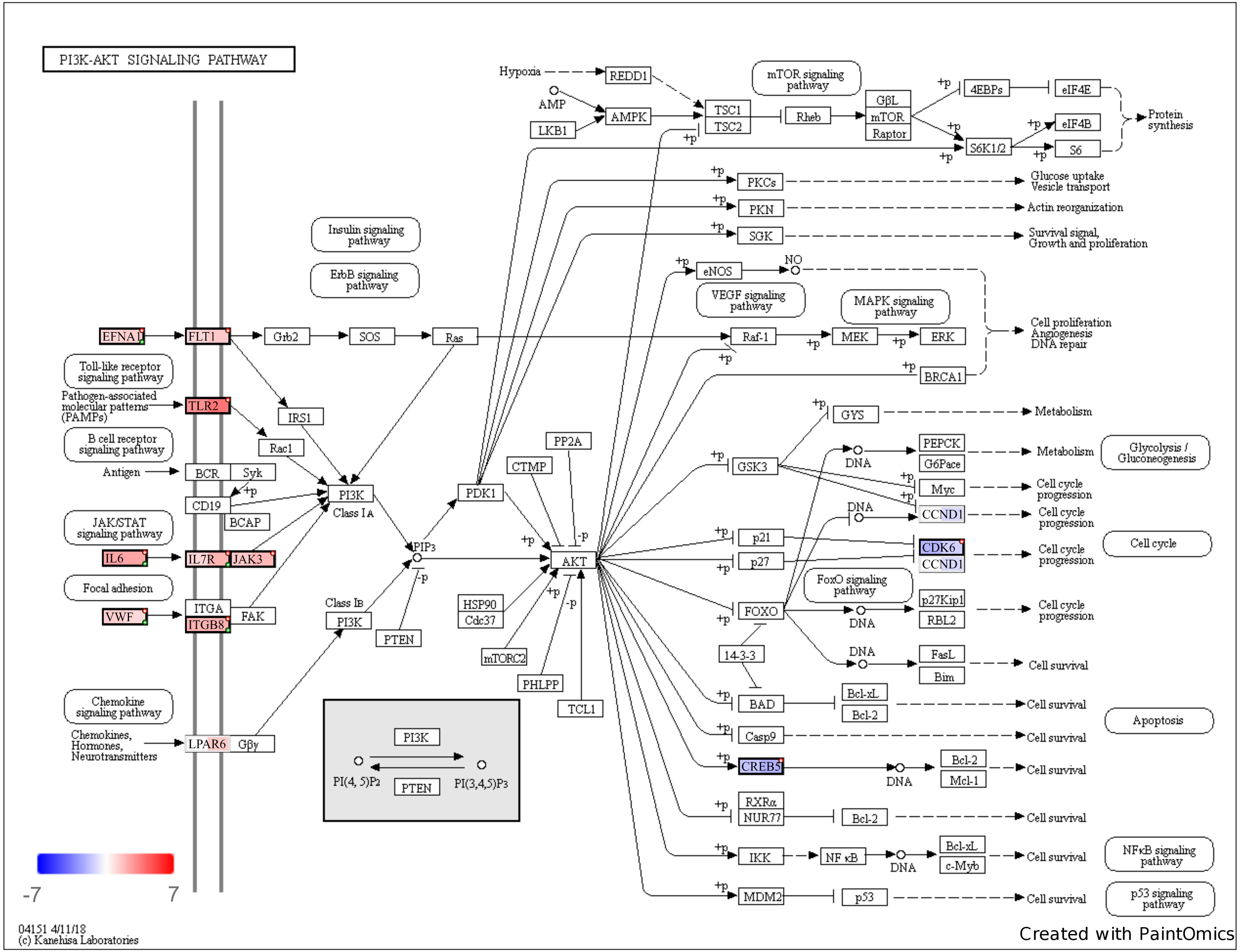

Supplementary dataset 2.3. Schematic view of NF-kappa B signaling pathway. Events leading to cell survival and induction of inflamation. Expressed genes in the hBMECs challenged with *S. pneumoniae* and adhesion lipoprotein (Lbp) are highlighted according to the scale (range of the fold change, LogFC values). Red shaded boxes indicate up-regulated genes and blue shaded boxes indicate down-regulated genes. Each box was automatically divided to show the gene expression, left half displays gene expression induced by *S. pneumoniae* while right half displays the gene expression evoked by Lbp. Genes induced in hBMECs by both treatments (*S. pneumoniae* or Lbp) are indicated as fully colored boxes and are marked with a red star in the right upper corner.
Pathways are taken from KEGG pathways (Kanehisa, M. and Goto, S.; KEGG: Kyoto Encyclopedia of Genes and Genomes. Nucleic Acids Res. 28, 27-30 (2000))

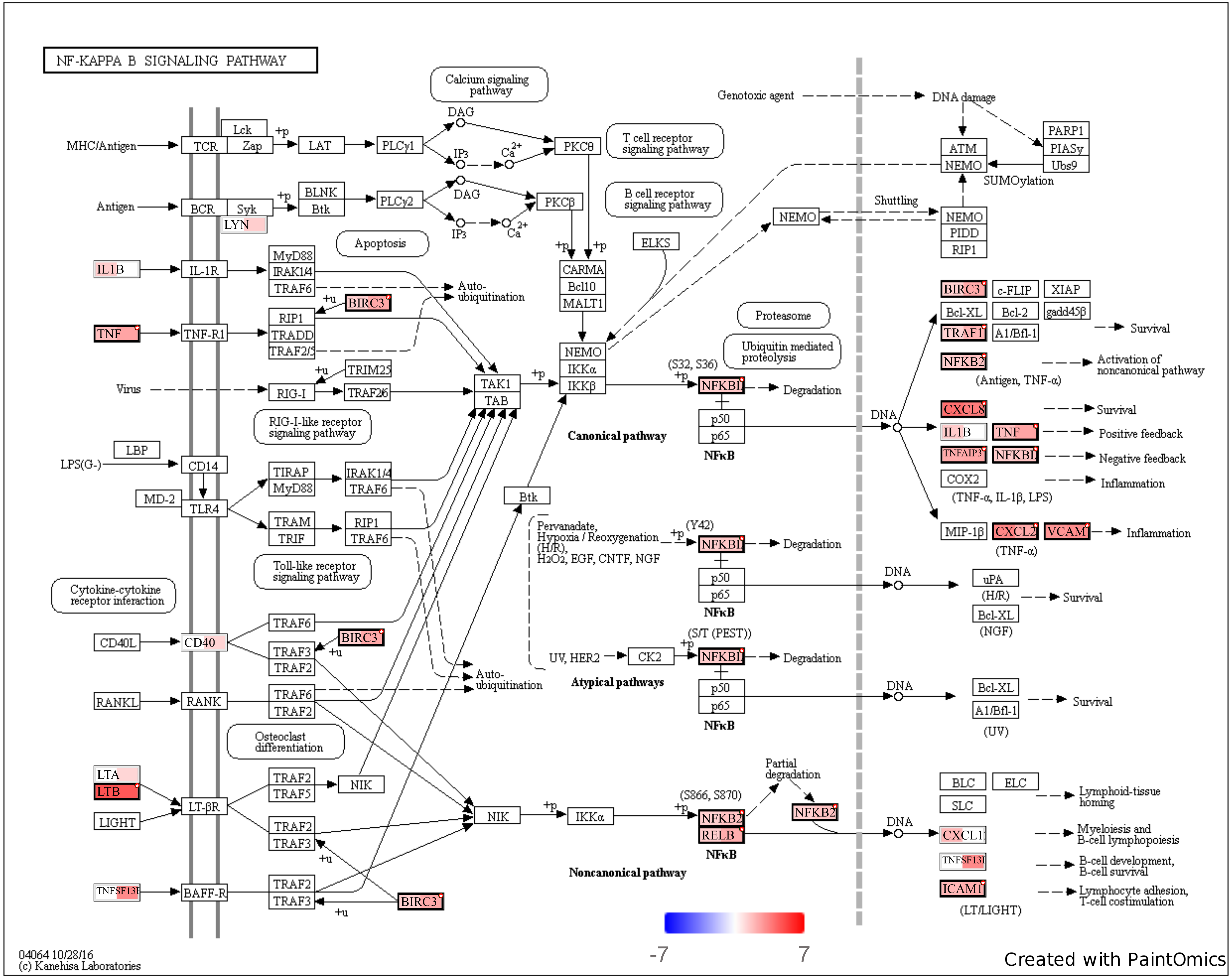

Supplementary dataset 2.4. Schematic view of Cytokine-cytokine receptor interaction. Pathway displaying interactions of cytokines that are intracellular regulators of cells enganged in innate and adaptive inflammatory host defenses. Expressed genes in the hBMECs challenged with *S. pneumoniae* and adhesion lipoprotein (Lbp) are highlighted according to the scale (range of the fold change, LogFC values). Red shaded boxes indicate up-regulated genes and blue shaded boxes indicate down-regulated genes. Each box was automatically divided to show the gene expression, left half displays gene expression induced by *S. pneumoniae* while right half displays the gene expression evoked by Lbp. Genes induced in hBMECs by both treatments (*S. pneumoniae* or Lbp) are indicated as fully colored boxes and are marked with a red star in the right upper corner. Pathways are taken from KEGG pathways (Kanehisa, M. and Goto, S.; KEGG: Kyoto Encyclopedia of Genes and Genomes. Nucleic Acids Res. 28, 27-30 (2000))

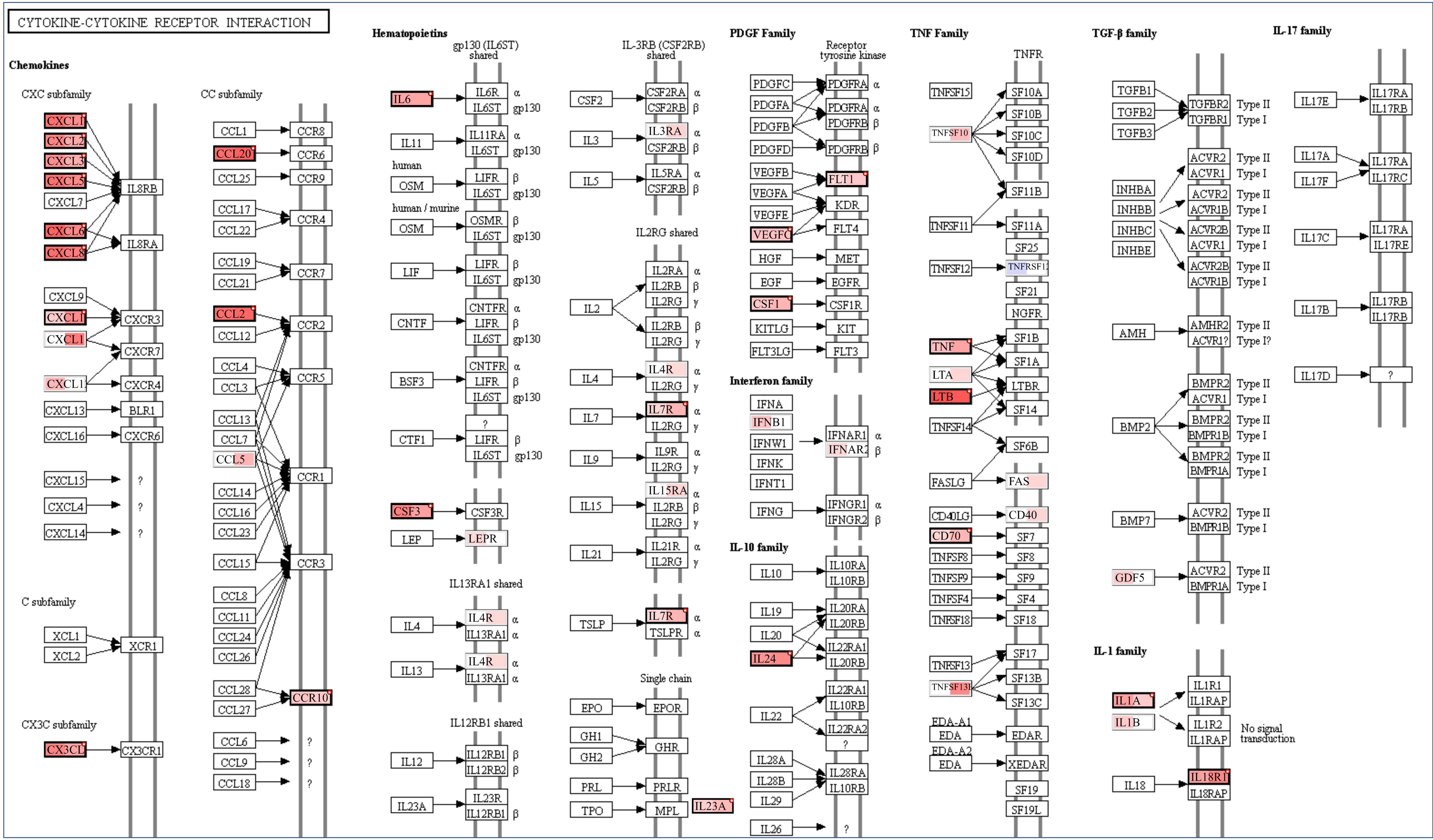

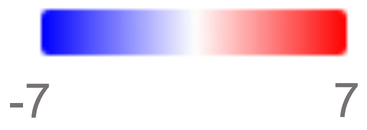

Supplementary dataset 2.5. Schematic view of Chemokine signaling pathway. Events required for recruitment of leukocytes to the site of inflammation. Expressed genes in the hBMECs challenged with *S. pneumoniae* and adhesion lipoprotein (Lbp) are highlighted according to the scale (range of the fold change, LogFC values). Red shaded boxes indicate up-regulated genes and blue shaded boxes indicate down-regulated genes. Each box was automatically divided to show the gene expression, left half displays gene expression induced by *S. pneumoniae* while right half displays the gene expression evoked by Lbp. Genes induced in hBMECs by both treatments (*S. pneumoniae* or Lbp) are indicated as fully colored boxes and are marked with a red star in the right upper corner.
Pathways are taken from KEGG pathways (Kanehisa, M. and Goto, S.; KEGG: Kyoto Encyclopedia of Genes and Genomes. Nucleic Acids Res. 28, 27-30 (2000))

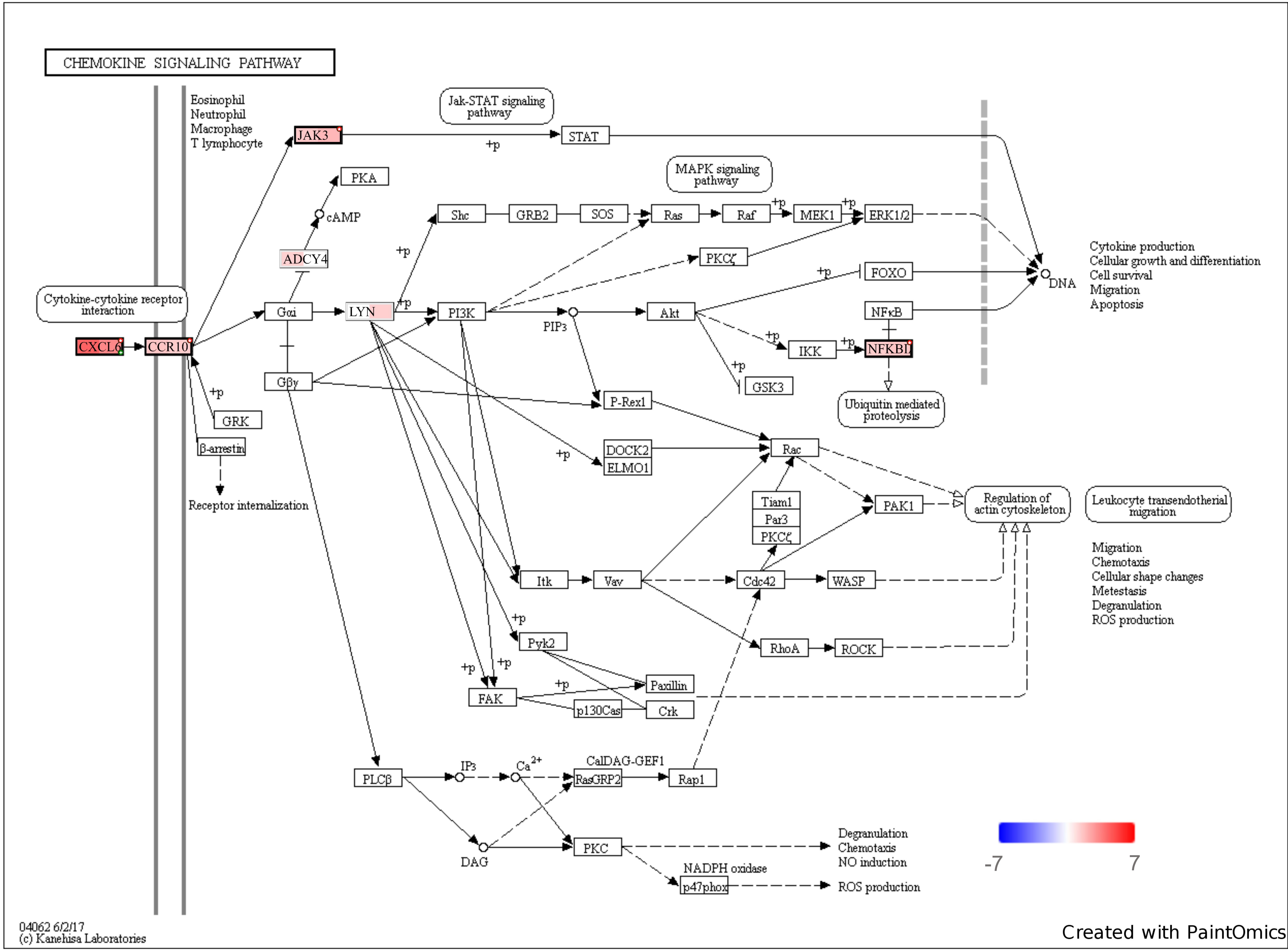

Supplementary dataset 2.6. Schematic view of TNF signaling pathway. Events occurring after activation of TNF leading to the leukocyte recruitment and activation of surface receptors. Expressed genes in the hBMECs challenged with *S. pneumoniae* and adhesion lipoprotein (Lbp) are highlighted according to the scale (range of the fold change, LogFC values). Red shaded boxes indicate up-regulated genes and blue shaded boxes indicate down-regulated genes. Each box was automatically divided to show the gene expression, left half displays gene expression induced by *S. pneumoniae* while right half displays the gene expression evoked by Lbp. Genes induced in hBMECs by both treatments (*S. pneumoniae* or Lbp) are indicated as fully colored boxes and are marked with a red star in the right upper corner.
Pathways are taken from KEGG pathways (Kanehisa, M. and Goto, S.; KEGG: Kyoto Encyclopedia of Genes and Genomes. Nucleic Acids Res. 28, 27-30 (2000))

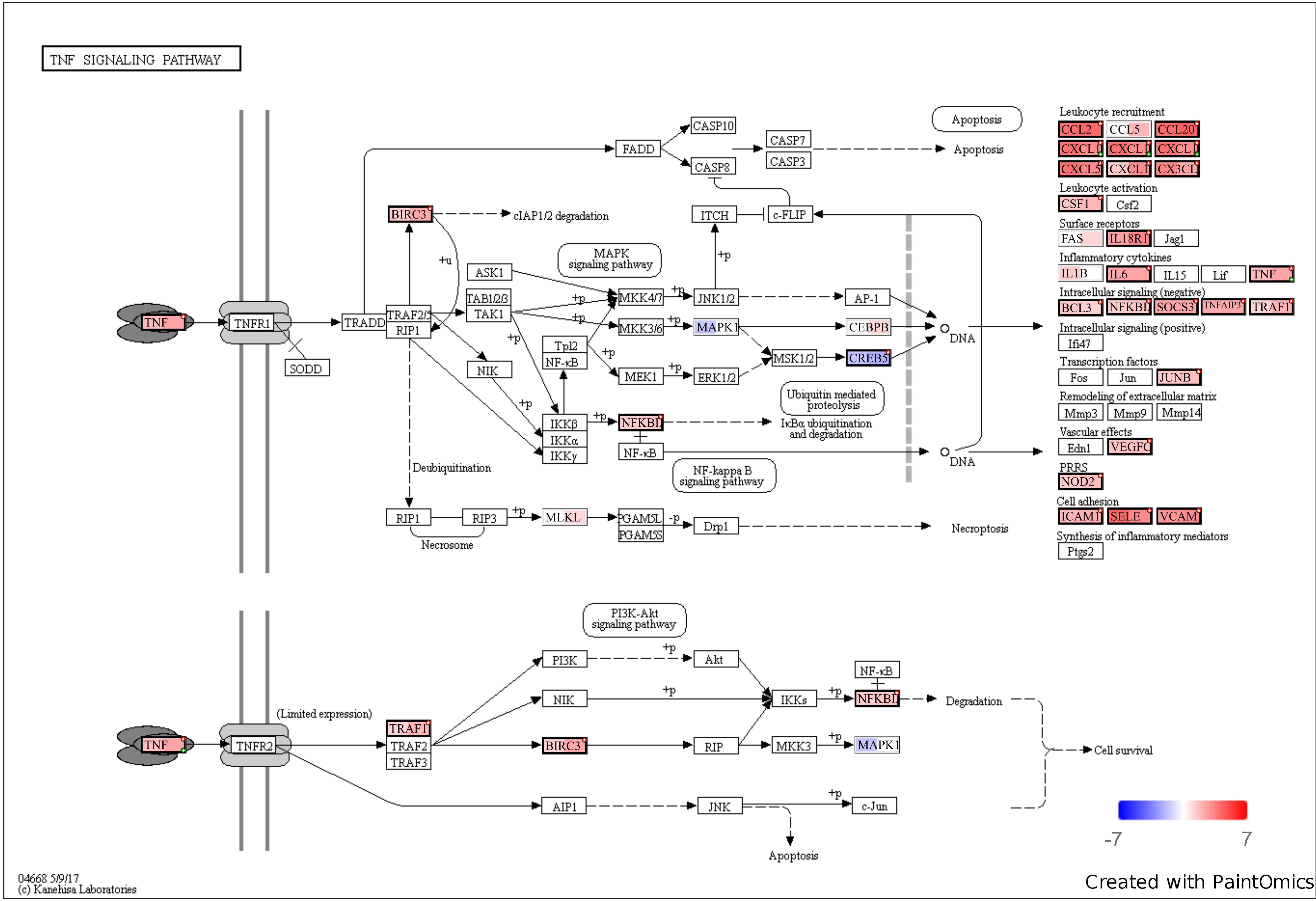

Supplementary dataset 2.7. Schematic view of the IL-17 signaling pathway. IL-17 family plays crucial roles in acute and chronic inflammatory responses having important roles in protecting the host against extracellular pathogens. Events involved in this pathway lead to neutrophil recruitment and inflammation. Expressed genes in the hBMECs challenged with *S. pneumoniae* and adhesion lipoprotein (Lbp) are highlighted according to the scale (range of the fold change, LogFC values). Red shaded boxes indicate up-regulated genes and blue shaded boxes indicate down-regulated genes. Each box was automatically divided to show the gene expression, left half displays gene expression induced by *S. pneumoniae* while right half displays the gene expression evoked by Lbp. Genes induced in hBMECs by both treatments (*S. pneumoniae* or Lbp) are indicated as fully colored boxes and are marked with a red star in the right upper corner.
Pathways are taken from KEGG pathways (Kanehisa, M. and Goto, S.; KEGG: Kyoto Encyclopedia of Genes and Genomes. Nucleic Acids Res. 28, 27-30 (2000))

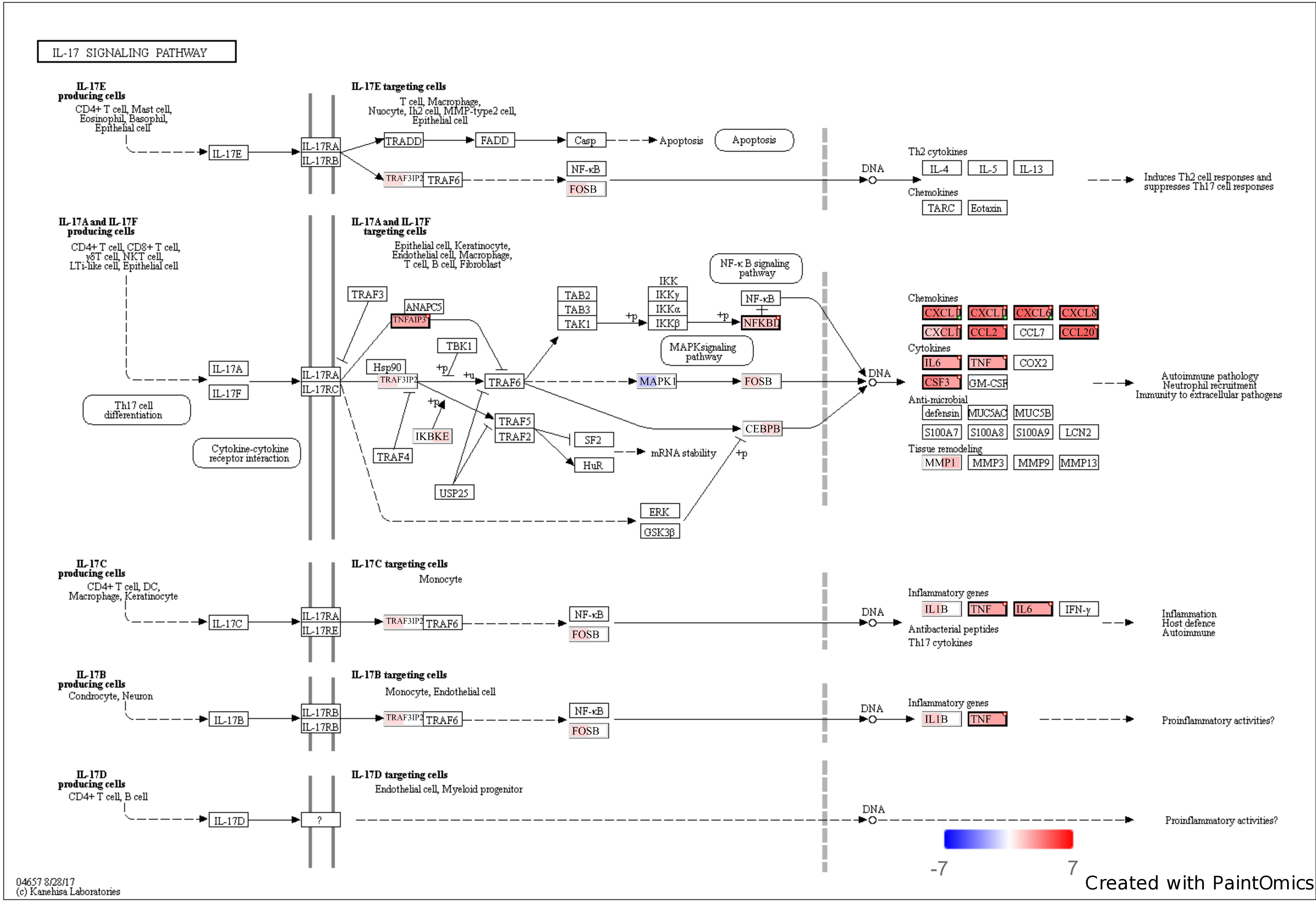

Created with PaintOmics

Supplementary dataset 2.8. Schematic view of Phagosome pathway. A phagosome is formed when specific receptors on the phagocyte surface recognize ligands on the particule surface. This pathway display events participating in the phagosome formation needed in phagocytosis. Expressed genes in the hBMECs challenged with *S. pneumoniae* and adhesion lipoprotein (Lbp) are highlighted according to the scale (range of the fold change, LogFC values). Red shaded boxes indicate up-regulated genes and blue shaded boxes indicate down-regulated genes. Each box was automatically divided to show the gene expression, left half displays gene expression induced by *S. pneumoniae* while right half displays the gene expression evoked by Lbp. Genes induced in hBMECs by both treatments (*S. pneumoniae* or Lbp) are indicated as fully colored boxes and are marked with a red star in the right upper corner.
Pathways are taken from KEGG pathways (Kanehisa, M. and Goto, S.; KEGG: Kyoto Encyclopedia of Genes and Genomes. Nucleic Acids Res. 28, 27-30 (2000))

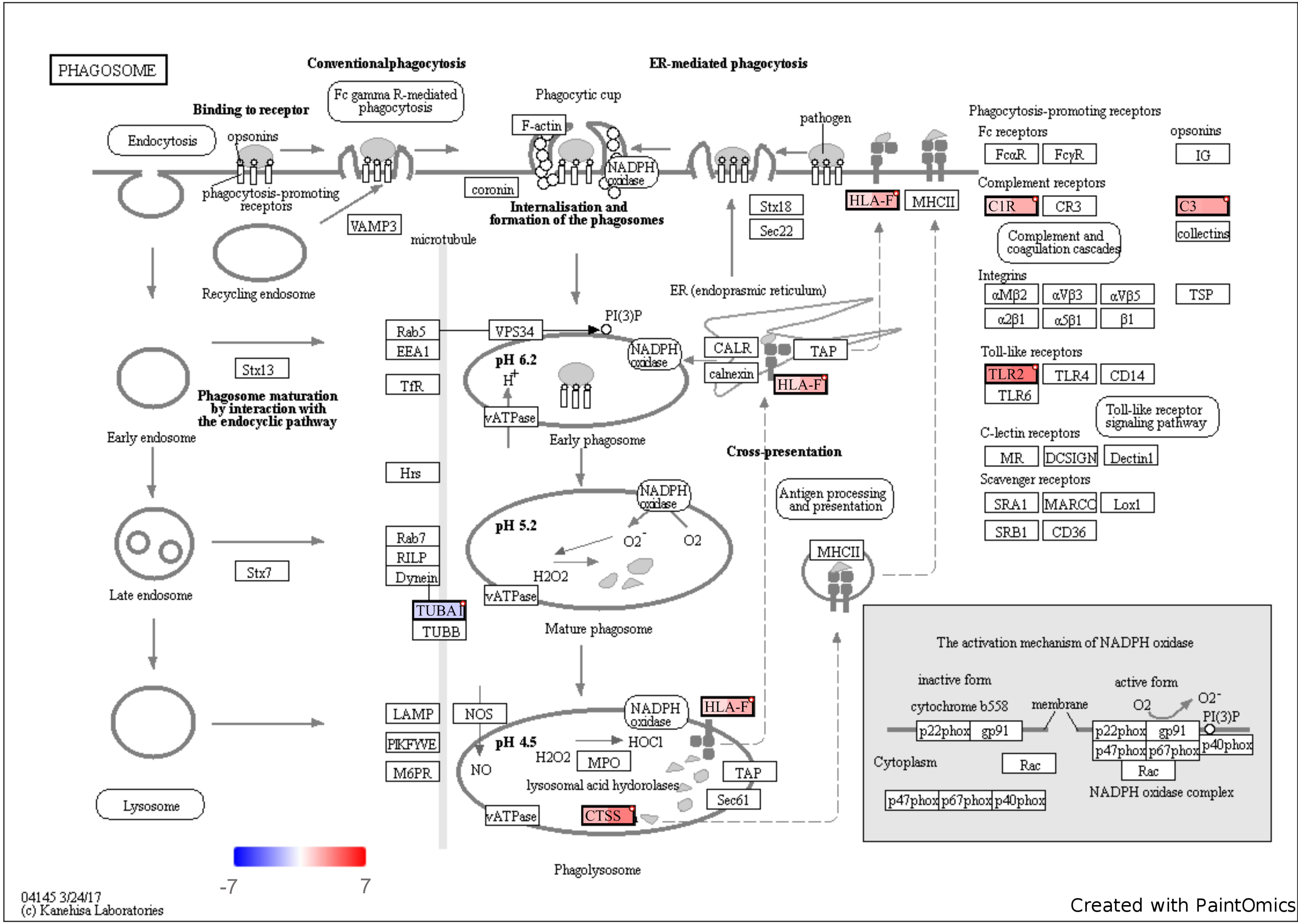

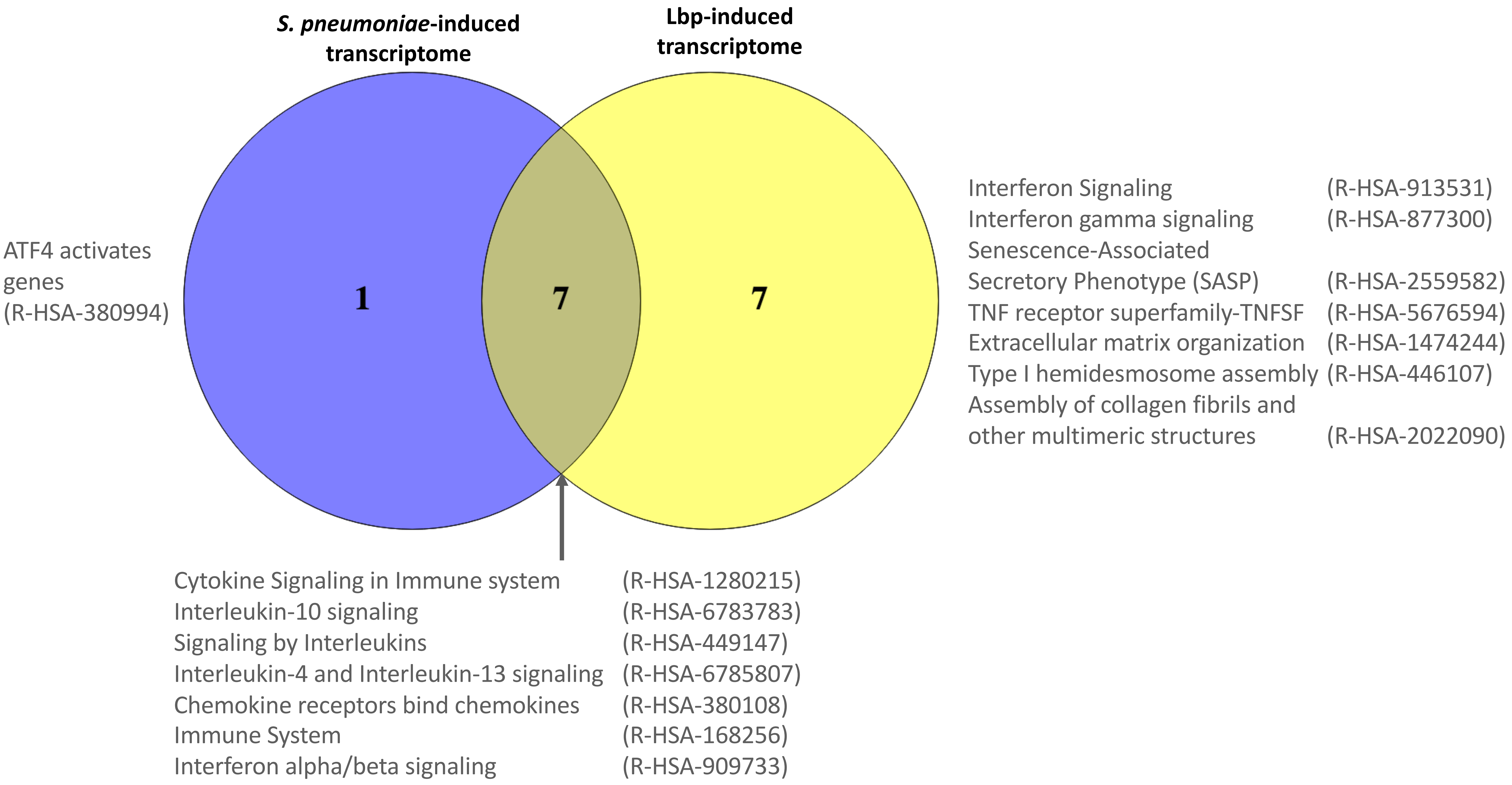

Supplementary dataset 2.9. Schematic view of signaling pathways identified in hBMECs transcriptomes. Venn diagram was created to show pathways identified in the *S. pneumoniae*-induced transcriptome (blue circle) and in the Lbp-induced transcriptome (yellow circle). Number of pathways identified in both treatments are shown as the intersection of both circles. Please note that common pathways were identified manually.

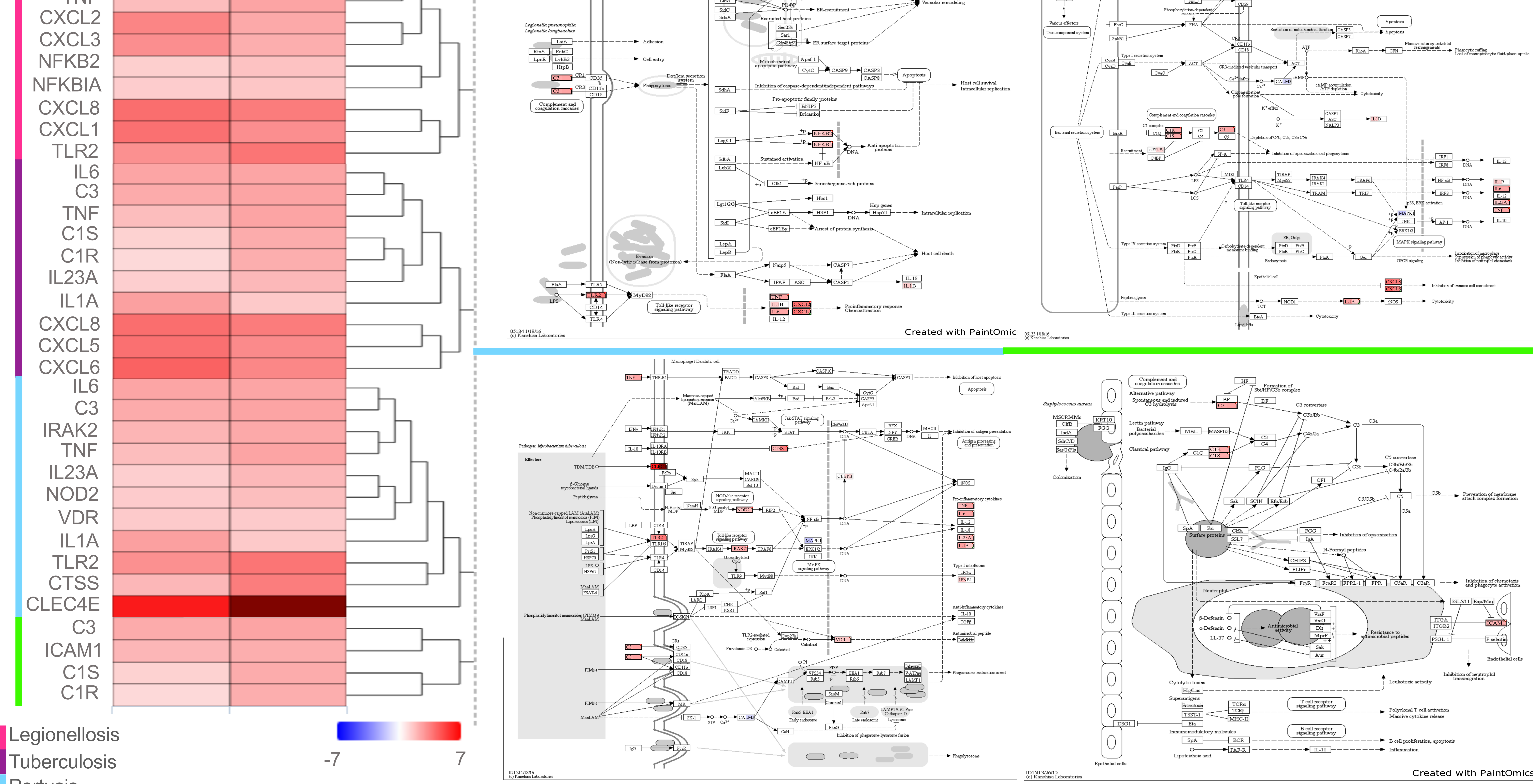

Supplementary dataset 2.10. Schematic view of DEGs induced by *S. pneumoniae* and Lbp that participate in signaling pathways related to bacterial infections. Panel A. Heatmap shows the gene expression of DEGs in hBMECs after treatmentwith *S. pneumoniae* and its protein Lbp. **Panel B.**Expressed genes in the hBMECs challenged with *S. pneumoniae* and adhesion lipoprotein (Lbp) are highlighted according to the scale (range of the fold change, LogFC values). Red shaded boxes indicate up-regulated genes and blue shaded boxes indicate down-regulated genes. Each box was automatically divided to show the gene expression, left half displays gene expression induced by *S. pneumoniae* while right half displays the gene expression evoked by Lbp. Genes induced in hBMECs by both treatments (*S. pneumoniae* or Lbp) are indicated as fully colored boxes and are marked with a red star in the right upper corner. Signaling pathways are indicated by colors: Legionellosis (Magenta color), tuberculosis (purple color), pertusis (light blue color) and S. aureus infection (green color). Pathways are taken from KEGG pathways (Kanehisa, M. and Goto, S.; KEGG: Kyoto Encyclopedia of Genes and Genomes. Nucleic Acids Res. 28, 27-30 (2000))
