# Supplementary material for: Transcriptomic analysis of human brain microvascular endothelial cells exposed to laminin binding protein (adhesion lipoprotein) and Streptococcus pneumoniae
Source: Sci Rep. 2021 Apr 12;11:7970. doi: 10.1038/s41598-021-87021-4 (PMC8041795; doi:10.1038/s41598-021-87021-4)
Supplement: Supplementary file 3 — Supplementary Information 3. [file 41598_2021_87021_MOESM3_ESM.pdf]

Transcriptomic analysis of human brain microvascular endothelial cells exposed to laminin binding protein (adhesion lipoprotein) and *Streptococcus pneumoniae*

Irene Jiménez-Munguía[1], Zuzana Tomečková[1], Evelína Mochnáčová[1], Katarína Bhide[1], Petra Majerová[2] and Mangesh Bhide[1,2]*

SUPPLEMENTARY FIGURES

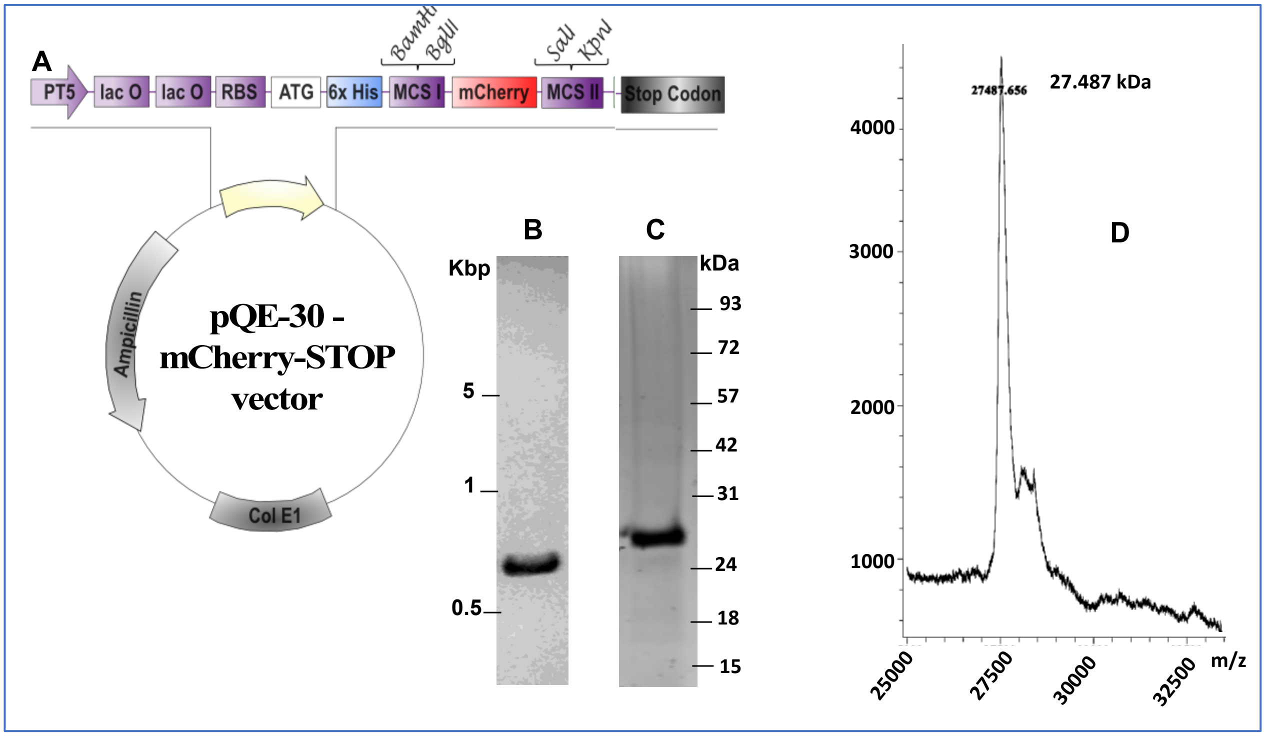

Supplementary information Fig. S1. Steps in production of recombinant forms of adhesion lipoprotein Lbp.

Panel A. pQE-30-mCherry-STOP plasmid (4880bp). Col E1 origin of replication, ampicillin resistance gene, PT5 T5 promoter, lac O lac operator, RBS ribosome binding site, ATG Start codon, 6xHis tag sequence, MCSI/MCSII multiple cloning sites, mCherry- red fluorescent protein that serve as stuffer, Stop codon. Backbone pQE-30 was obtained from Qiagen, Germany. **Panel B**, amplicon (717 bp + 20 bp overhangs) of the gene coding fragment of Lam gene (encoding Laminin binding protein – Lbp an adhesion lipoprotein – Spr0906) resolved on agarose gel. **Panel C**, purified recombinant Lbp separated with SDS-PAGE (~27.5 kDa). **Panel D**, Molecular mass of the recombinant Lbp confirmed with MALDI-TOF/MS (24.487 kDa). Please note that, the sequence homology between Lbp of the strain used in this study and TIGR4 strain is 99.16% (one amino acid change at 271 S to A; Lbp of TIGR4 Genbank accession number WP_000744528).

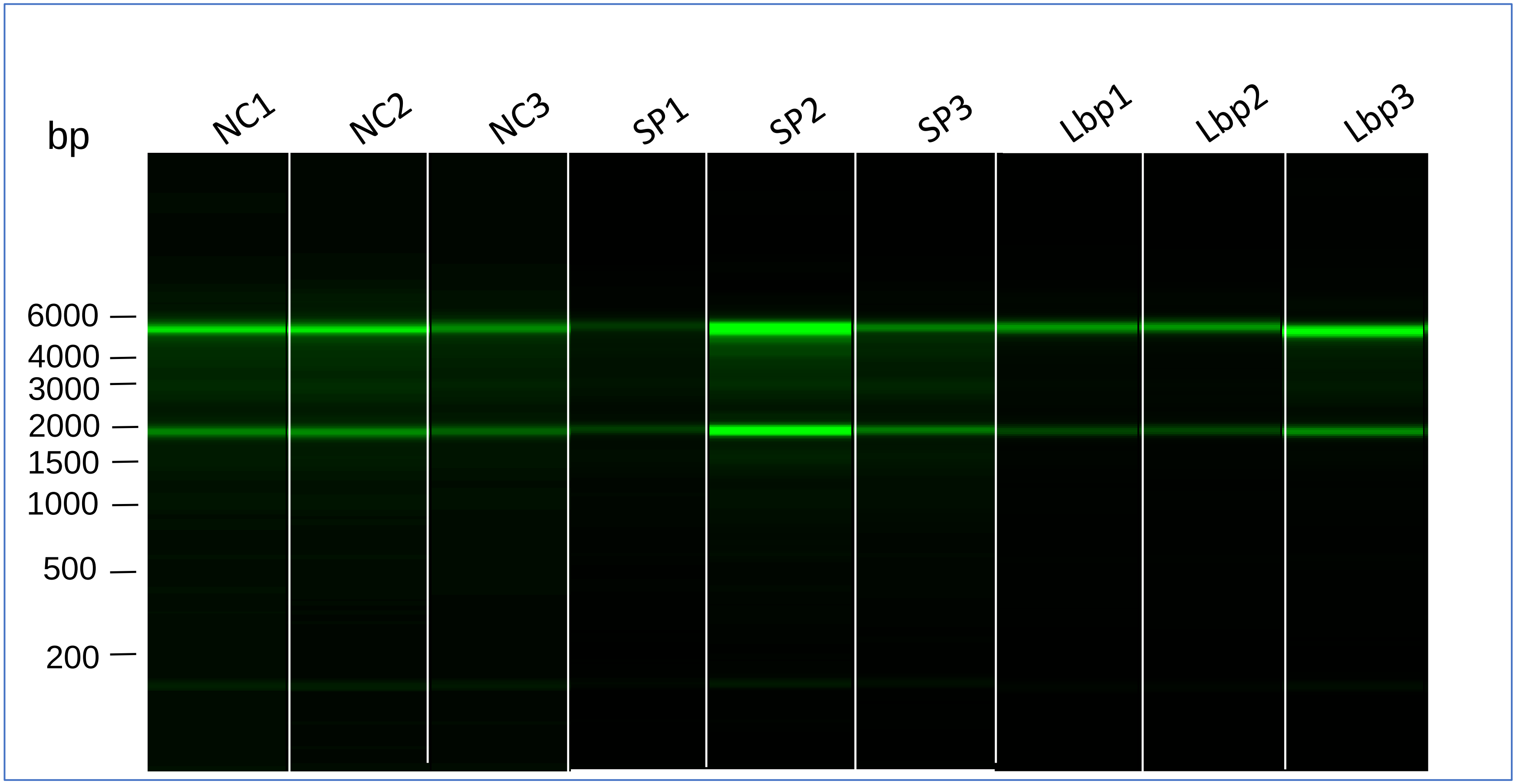

Supplementary information Fig. S2. Integrity of RNA. RNA isolated from non-induced hBMECs (negative control, NC1 to 3) and hBMECs incubated either with intact *S. pneumoniae* (SP1 to 3) or adhesion lipoprotein Lbp (Lbp-1 to 3).

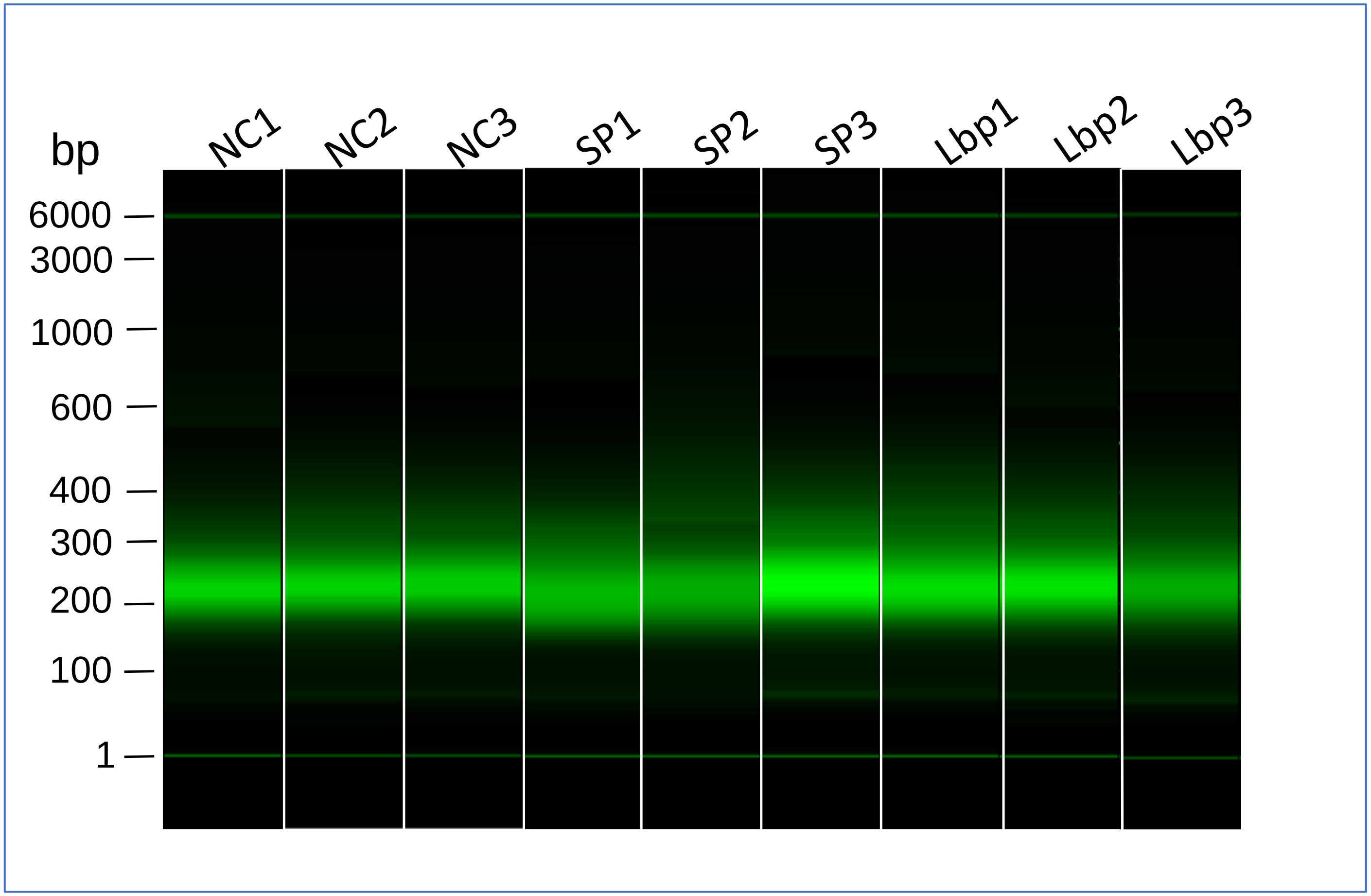

Supplementary information Fig. S3. Capillary electrophoresis for the quality control of cDNA libraries prepared for RNA-seq. Nine cDNA libraries for sequencing were prepared with QuantSeq 3' mRNA kit corresponding to non-induced hBMECs (negative control, NC1 to 3) and hBMECs incubated either with intact *S. pneumoniae* (SP1 to 3) or adhesion lipoprotein Lbp (Lbp-1 to 3). Ideal fragment size should be between 150-300 bp.

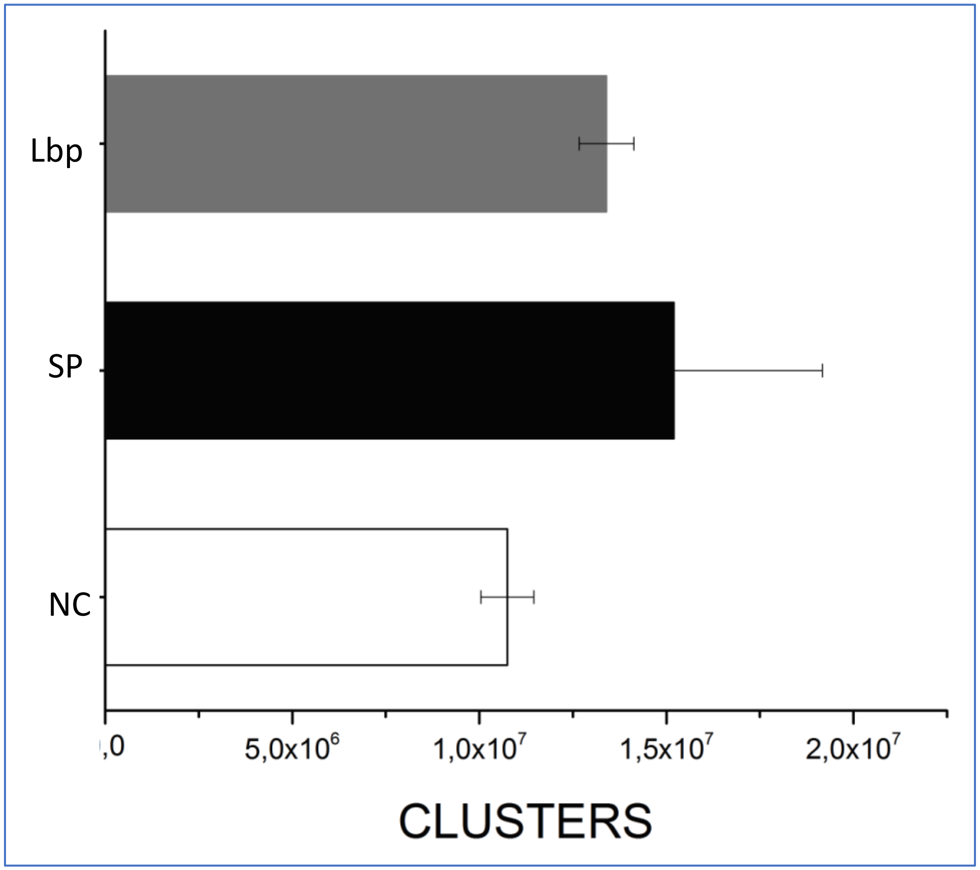

Supplementary Fig. S4. Clusters identified in the RNA-seq analysis. Raw reads determined from RNA sequencing of non-induced hBMECs (negative control, NC) or incubated either with *S. pneumoniae* (SP) or adhesion lipoprotein Lbp. Standard deviations were calculated from three biological replicates.

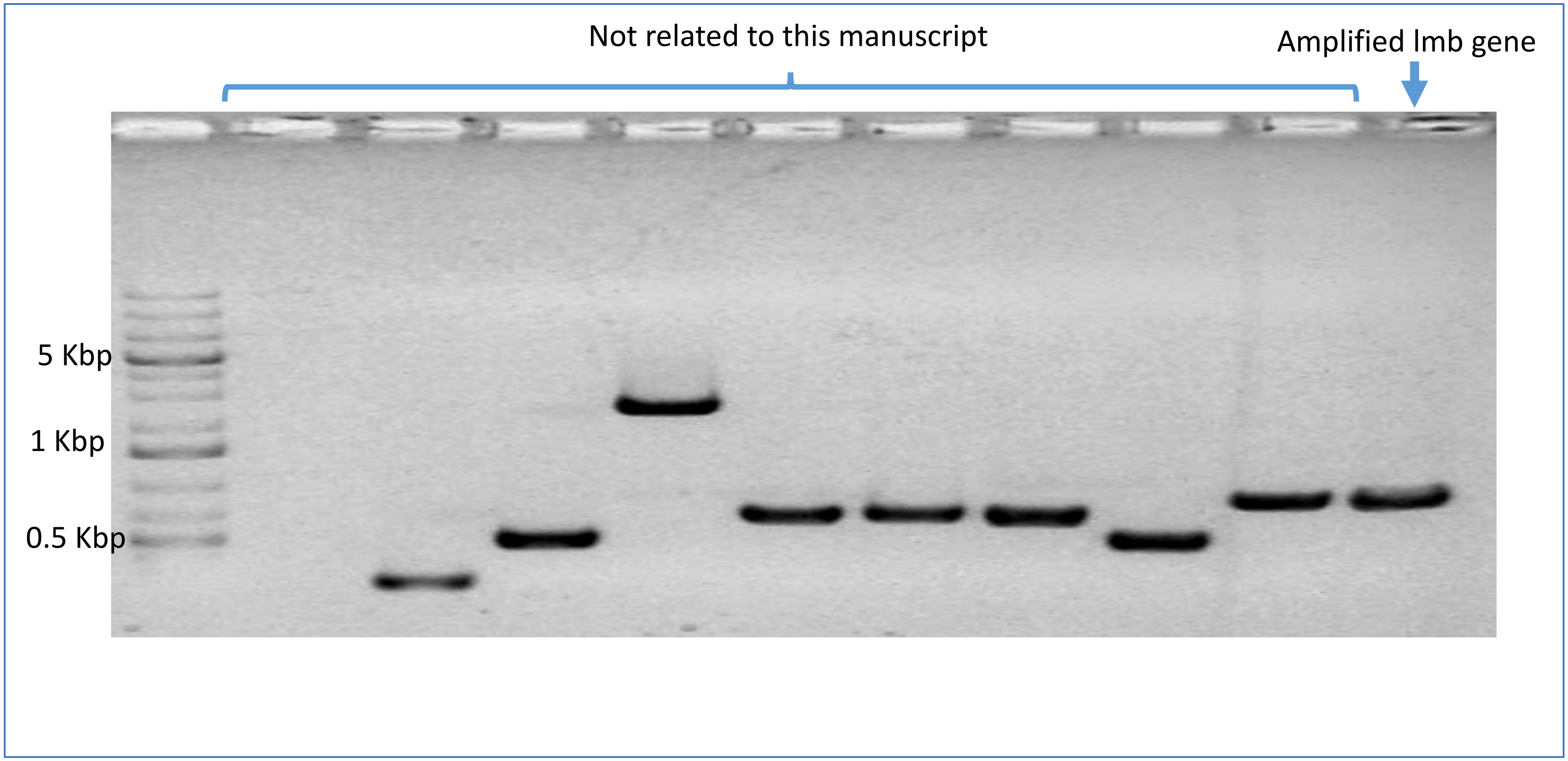

Supplementary Fig. S5. Original photograph of the agarose gel used to make panel B of Supplementary Fig. S1

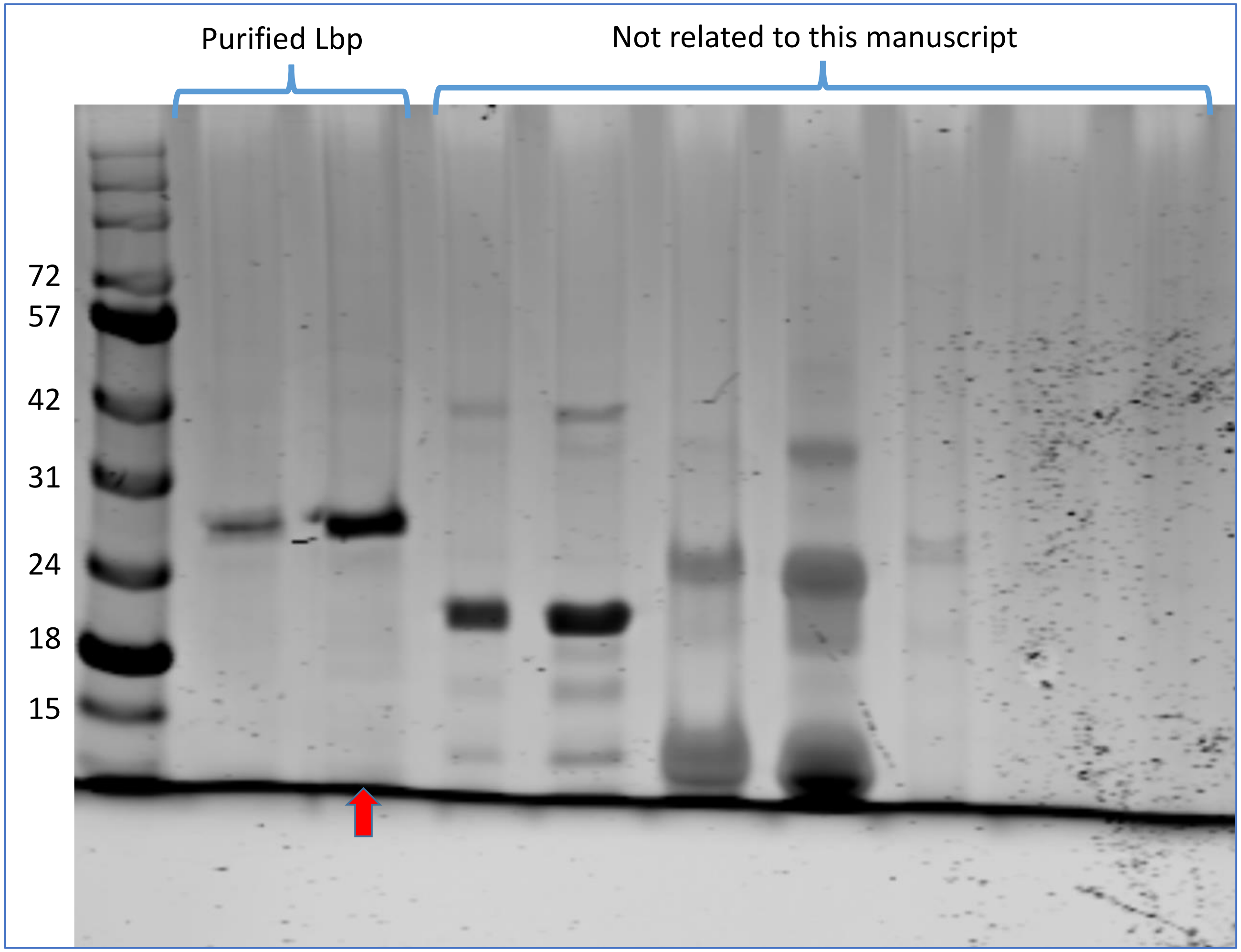

Supplementary Fig. S6. Original photograph of the polyacrylamide gel (SDS_PAGE) used to make panel C of Supplementary Fig. S1.

Arrow indicates the lane used in panel C of of Supplementary Fig. S1.

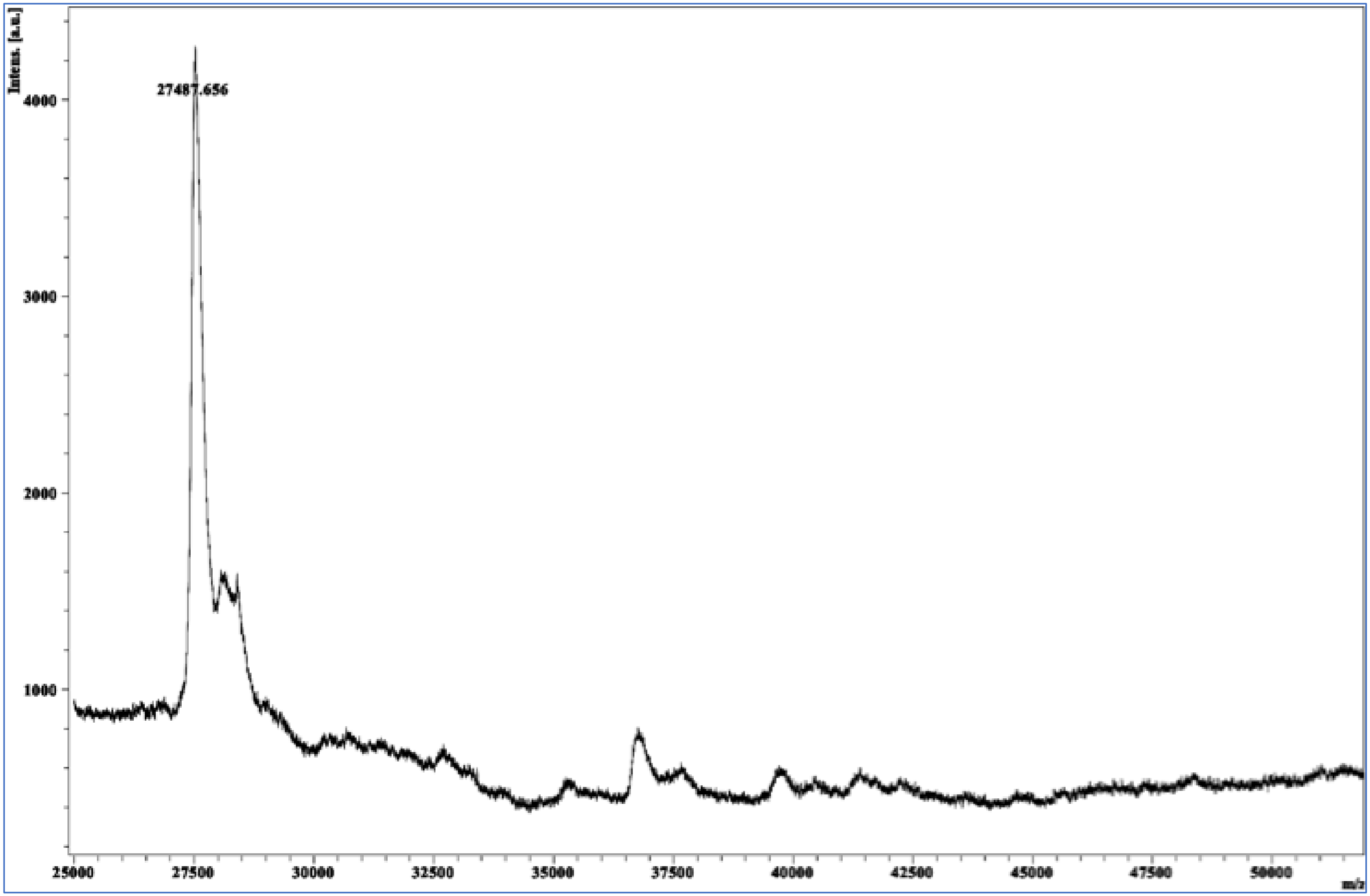

Supplementary Fig. S7. Original spectrum (MALDI-TOF-MS) used to make panel D of Supplementary Fig. S1.

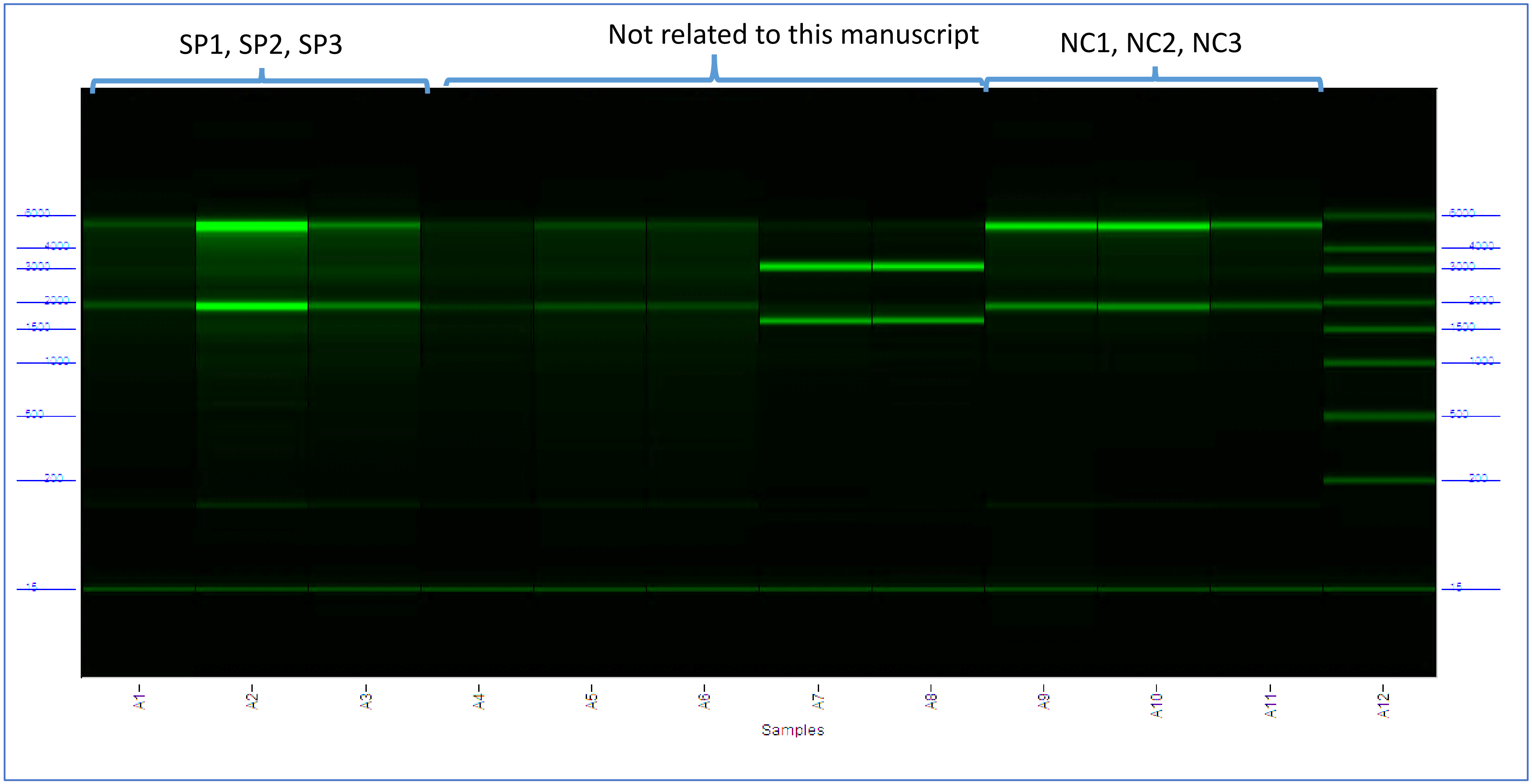

Supplementary Fig. S8. Original photograph used to make Supplementary Fig. S2.

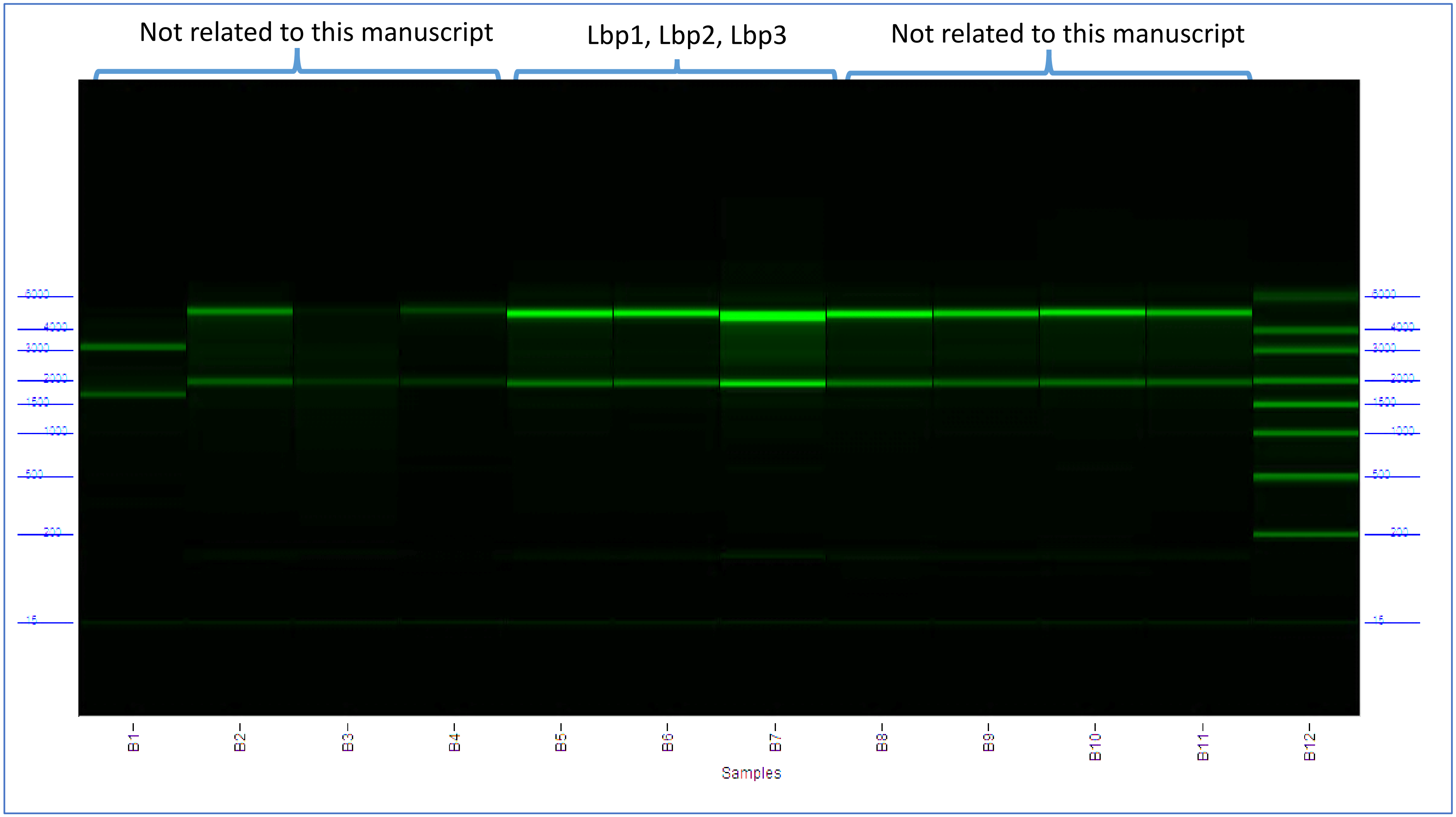

Supplementary Fig. S8. Original photograph used to make Supplementary Fig. S2.

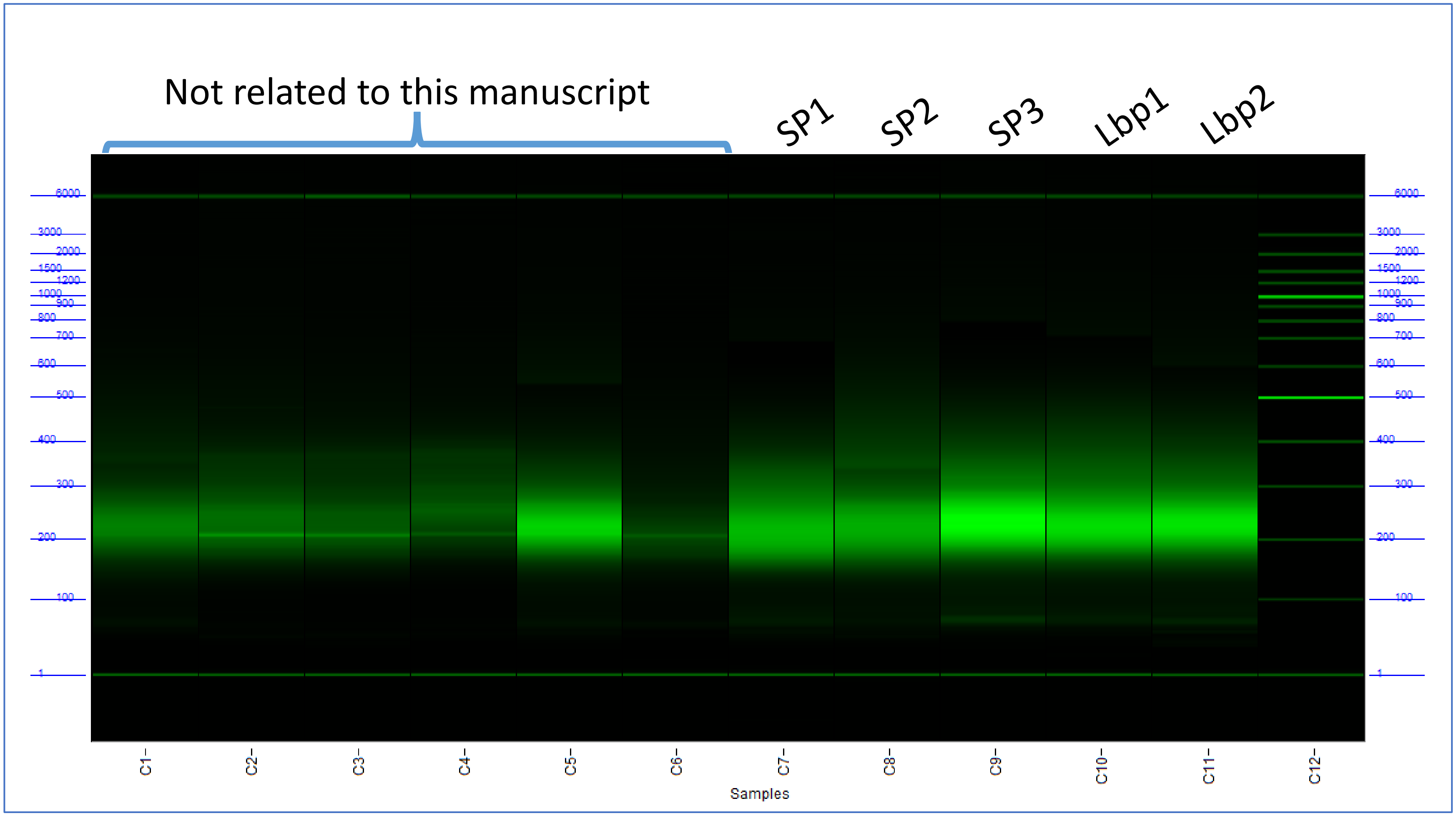

Supplementary Fig. S9. Original photograph used to make Supplementary Fig. S3.

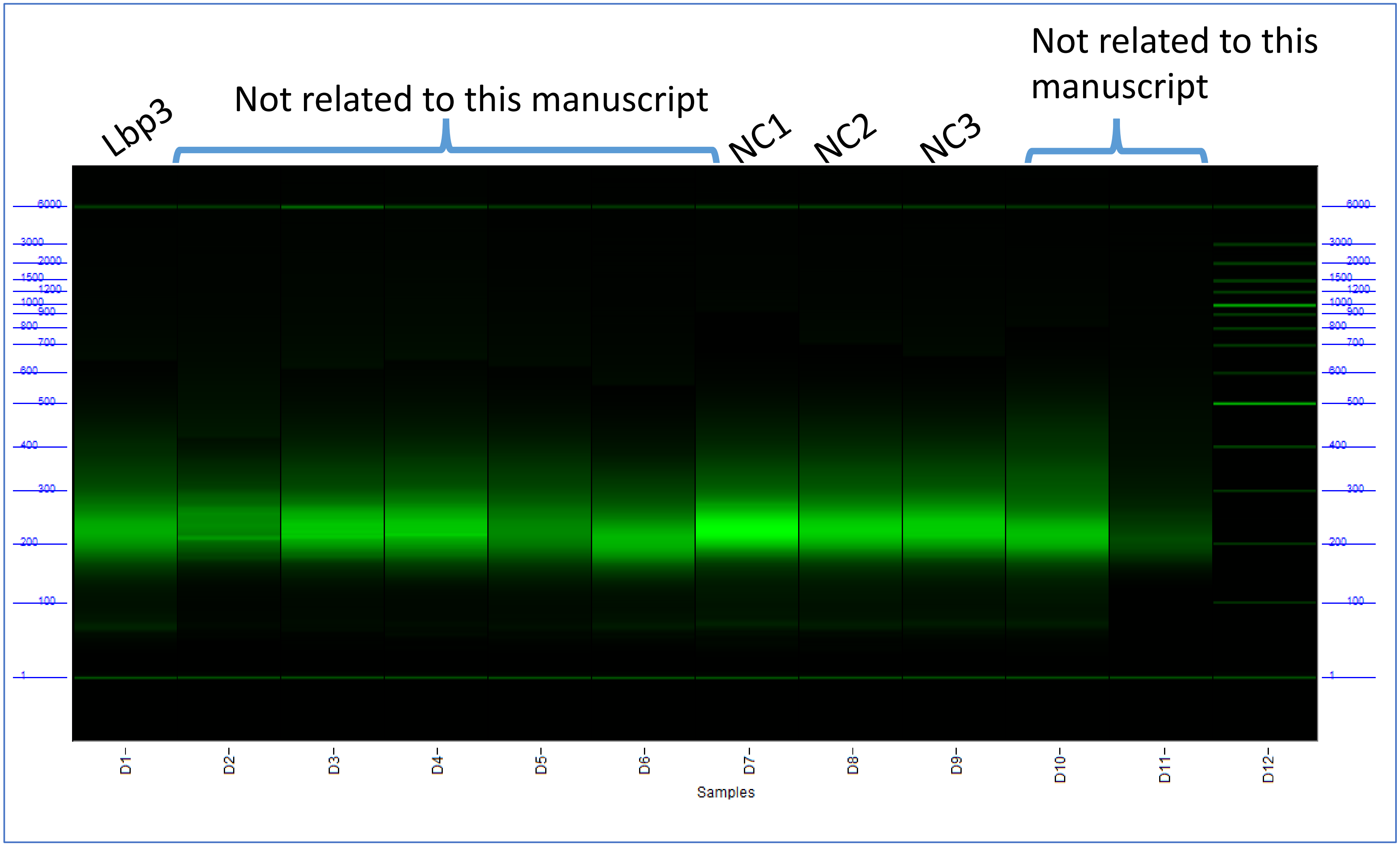

Supplementary Fig. S10. Original photograph used to make Supplementary Fig. S3.

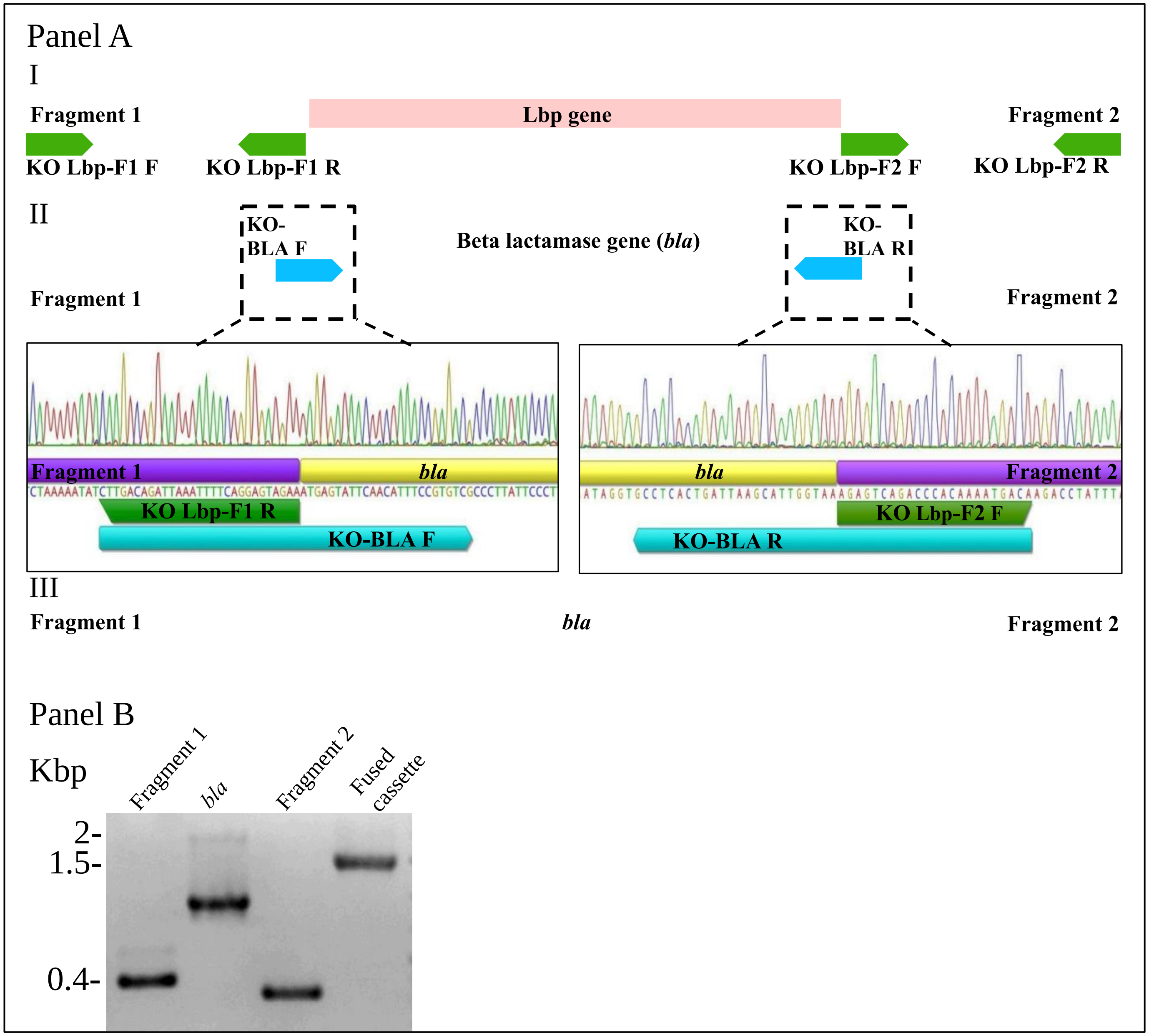
Panel A
I
Fragment 1
Lbp gene
Fragment 2
KO Lbp-F1 F
KO Lbp-F1 R
KO Lbp-F2 F
KO Lbp-F2 R
II
KO-
BLA F
Beta lactamase gene (bla)
KO-
BLA R
Fragment 1
Fragment 2
Fragment 1
bla
KO Lbp-F1 R
KO-BLA F
bla
Fragment 2
KO Lbp-F2 F
KO-BLA R
III
Fragment 1
bla
Fragment 2
Panel B
Kbp
Fragment 1
bla
Fragment 2
Fused
cassette
2-
1.5-
0.4-

Supplementary Fig. S11. Steps involved in deletion of Lbp gene

Panel A- Schematic representation of construction of fused cassette used to knock-out *lbp* gene. 351 bp upstream (fragment 1) and 300 bp (fragment 2) downstream to *lbp* gene were amplified using primers depicted in I (Green bars). *bla* gene encoding beta-lactamase was amplified from pLEXY I-ble3 plasmid (Jena Bioscience, Germany) using primers shown in II (KO-BLA F and KO-BLA R). Sequences of all primers are in table 1.

Fragment 1, bla and fragment 2 were fused with overlap extension PCR (OE-PCR). As depicted in II, KO-BLA F and KO-BLA R primers (used to amplify *bla*) have 29 bp and 23 bp overlaps to fragment 1 and fragment 2, respectively. These overlaps ensure fusion of three fragments during the first round of OE-PCR in direction: *5'- fragment 1- bla -fragment 2 -3'.* Fused cassette (as depicted in III) was amplified using PCR-product from first step of OE-PCR and primers KO-Lbp-F1-F and KO-Lbp-F2-R. Fused cassette was sequenced to corroborate proper fusion.

Panel B- Agarose gel depicting amplicons of the fragment 1, *bla* gene, fragment 2 and full fused cassette after second round of OE-PCR.

Confirmation of the deletion in mutant clone was performed by sequencing. Chromatograms of the fused regions (DNA amplified from mutant clone) are presented in II of panel A.

**Supplementary information Table S1. Comparison analysis of the biological processes identified in the induced hBMECs transcriptomes**						
No.	Reactome Server				PaintOmics	
	S. pneumoniae-induced hBMECs transcriptome		Lbp-induced hBMECs transcriptome		Both induced hBMECs transcriptome	
	ID	Pathway name	ID	Pathway name	ID	Pathway name
1	R-HSA-1280215	Cytokine Signaling in Immune system	R-HSA-1280215	Cytokine Signaling in Immune system	hsa04668	TNF signaling pathway
2	R-HSA-6783783	Interleukin-10 signaling	R-HSA-6783783	Interleukin-10 signaling	hsa04064	NF-kappa B signaling pathway
3	R-HSA-449147	Signaling by Interleukins	R-HSA-449147	Signaling by Interleukins	hsa04514	Cell adhesion molecules (CAMs)
4	R-HSA-6785807	Interleukin-4 and Interleukin-13 signaling	R-HSA-6785807	Interleukin-4 and Interleukin-13 signaling	hsa04062	Chemokine signaling pathway
5	R-HSA-380108	Chemokine receptors bind chemokines	R-HSA-168256	Immune System	hsa04060	Cytokine-cytokine receptor interaction
6	R-HSA-168256	Immune System	R-HSA-913531	Interferon signaling	hsa04657	IL-17 signaling pathway
7	R-HSA-909733	Interferon alpha/beta signaling	R-HSA-380108	Chemokine receptors bind chemokines	hsa04151	PI3K-Akt signaling pathway
8	R-HSA-380994	ATF4 activates genes	R-HSA-909733	Interferon alpha/beta signaling	hsa04145	Phagosome
9			R-HSA-877300	Interferon gamma signaling		
10			R-HSA-2559582	Senescence-Associated Secretory Phenotype (SASP)		

11			R-HSA-5676594	TNF receptor superfamily (TNFSF) members mediating non-canonical NF-kB pathway		
12			R-HSA-1474244	Extracellular matrix organization		
13			R-HSA-446107	Type I hemidesmosome assembly		
14			R-HSA-2022090	Assembly of collagen fibrils and other multimeric structures		

Supplementary information Table S2. **Primers used to produce recombinant forms of the Lbp**

Protein/(Gene)	Sequence used to design primers	Primer	Sequence (5´- 3´)	Amplicon length (bp)
Adhesion lipoprotein(*lmb)*	AE007317.1*: nt891426 to nt892361	*spr0906*- sense	TATAGATCTGGTGACTTGAATGATGTTCGG	717
		spr0906- antisense	TTTGTCGACGGTCTTGTCATTTTGTGGGTC	

*shows Genbank accession number followed by nucleotide positions spanning the gene. Restriction sites are depicted with underlined nucleotides. AGATCT – *Bgl*II and GTCGAC - *Sal*I

**Supplementary information Table S3. Vector specific primers used to confirm presence of insert gene in transformants**	
Primer	Sequence (5´- 3´)
UA Insertom F	CGCATCACCATCACCATCACG
UA Insertom R	ACCAAATTGGGACAACACCAGTG

SUPPLEMENTARY METHODS

Supplementary method S1

Human brain microvascular endothelial cell (hBMECs) culture.

hBMEC (D3 cell line) was obtained from Merck/Millipore (Prague, Czech Republic). Cells were cultured in 25-mL cell culture flask coated with collagen type I (Sigma, USA) in EBM-2 medium (Lonza, UK) containing 10% FBS, gentamycin, 1.4 µM hydrocortisone (Sigma), 5 µg/mL ascorbic acid, 10 mM HEPES and 1 ng/mL bFGF (Sigma). Cells were incubated at 37°C in a humid atmosphere of 5% CO_2 until confluence. Cells were passaged on 6-well cell culture plate covered with collagen type I (Sigma, USA) in EBM-2 medium (Lonza, UK). Cells from the monolayer (6th passage) were used in the experiment. Cells were either harvested for RNA isolation (non-induced cell control) or incubated with live *S. pneumoniae* or Lbp.

Supplementary method S2

Selection and culture of clones for recombinant protein production. For clonal selection, the experimental conditions were kept exactly as described in our recent publication[1]. The gene fragment encoding Lbp was amplified by PCR from genomic DNA. Detailed information on primer, amplicon length and restriction enzymes used are presented in **Supplementary Table S2 online**. Amplified fragment was digested with restriction enzymes *Bgl*II and SalI (Thermo Fisher Scientific, Slovakia), as per the manufacturer's instruction. Digested fragments were ligated into pQE-30-mCherry-STOP plasmid (**Supplementary Fig. S1 online**). Please note that in this vector mCherry serves as stuffer sequence, which is cut out during the digestion of vector with restriction enzymes. Ligation mix contained 2µl of ligation buffer, 2µl of PEG8000, 0.5 U of the T4 ligase, amplicon and plasmid (50 ng) in molar ratio of 10:1, and water up to 20 µl. Ligation was performed at 22°C for 1 hr. Ligation mix was purified using NucleoSpin (Macherey-Nagel, Germany) and transformed into *E. coli* M15 strain (Qiagen, Germany). Transformants were selected from LB agar plates (lysogeny broth, Sigma; supplemented with 2% bacteriological agar, 1% glucose, 25 µg/mL kanamycin and 50 µg/mL carbenicillin).

Presence of encoding gene in transformants was confirmed by sequencing (vector specific primers UA Insertom F and R, presented in **supplementary table S3 online**).

A single colony carrying *lmb* gene was cultivated in Terrific broth (TB) (15 g/L tryptone, 30 g/L yeast extract, 12.5 g/L NaCl, 2.5 g/L $MgCl_2$/$MgSO_4$, 100 µL/L metal mix, 7.5 mL/L glycerol) supplemented with 1% glucose, 50 µg/mL carbenicillin and 25 µg/mL kanamycin until OD_{600}= 6. Bacterial cells were pelleted (centrifugation at 6,000 × g for 10 minutes) and resuspended in fresh TB medium without glucose. Protein expression was induced with 1 mM IPTG (Fermentas, Slovakia) at 20 °C for 20 hrs.

Supplementary method S3

Purification of the Lbp. Purification of Lbp was performed as described in our publication[1]. After induction, cells were pelleted (17,880 × g for 10 minutes) and lysed in lysis buffer (0.03 M Na_2HPO_4, 0.5 M NaCl, 0.001% Tween 20, 10% glycerol, 1x protease inhibitor cocktail, Sigma-Aldrich) with four freeze-thaw cycles followed by sonication on ice (2 cycles; 30-s pulses, 100% amplitude). Proteins were purified with nickel affinity chromatography (Ni-NTA agarose beads, ABT agarose Spain) as per manufacturer´s instructions. Briefly, Ni-NTA beads were equilibrated with the buffer (50 mM Na_2HPO_4, 300 mM NaCl, 0.001% Tween 20, pH 8). Beads were then incubated in the lysate for 2 hr at 8°C with constant rotation (140 rpm). Unbound proteins were washed with washing buffer (50 mM Na_2HPO_4, 300 mM NaCl, 0.001% Tween 20, 20 mM imidazole, pH 8) for 5 times and protein were eluted in elution buffer (50 mM Na_2HPO_4, 300 mM NaCl, 250 mM imidazole, pH 8).

Protein was immediately subjected to gel filtration (Sephadex G25, 30 ml column, *in-house* prepared) on ÄKTApurifier (GE-healthcare, 2 ml/min flow, max 0.45 MPa pressure) to remove imidazole and accomplish the buffer exchange. The buffer used for exchange was 50 mM Na2HPO4 buffer pH 7.0. 50 mM Na_2HPO_4 buffer pH 7.0 was added to the eluted protein to adjust the Nacl concentration between 0.1 - 0.2 M and protein was loaded on modified-polylysine resin (high capacity endotoxin removal resin, Thermo-scientific) packed in 1 ml column (*in-house* column packing). Conditions for this chromatography performed on ÄKTApurifier were: both start and elution buffers (isocratic condition) - 25mM sodium phosphate buffer

containing 0.1 M NaCl pH 7.0 (prepared in endotoxin free water), flow rate - 0.15 ml/min, pressure limit - 0.25 MPa maximum, sample load - 2 ml. Please note that eluate was collected in biosphere endotoxin free tubes (Sarsted, Germany) and protein was stored immediately in several aliquots until use.

Supplementary method S4

SDS-PAGE and MADLI-TOF

Whole cell lysate of *E.coli*, flow-through from nickel affinity chromatography and Lbp eluted after endotoxin removal were separated on SDS-PAGE. Briefly, the protein samples (5 µl each) were mixed with lithium dodecyl sulfate sample buffer (4X LDS sample buffer, Invitrogen, Slovakia) as per manufacturer's instructions and incubated at 72°C for 15 min. Electrophoresis was carried out at 40 mA in 1 x running buffer (20 x NuPAGE MOPS SDS running buffer, Invitrogen) until the dye reached the bottom of the gel (10% Bis-Tris Polyacrylamid 12 well gel, Invitrogen). Protein were stained with silver staining kit as per manufacturer's instructions (Bio-Rad).

For MALDI-TOF, 0.8 µl of the purified protein (after endotoxin removal) was mixed with 0.8 µl sDHB matrix (Bruker Daltonics, Germany; sDHB dissolved up to saturation in TA50 (50:50 [v/v] acetonitrile : 0.1% TFA in water). 1 µl of the protein-matrix mix was spotted on the ground-steel plate (Bruker Daltonics) and allowed to air dry. Acquisition was performed in flexControl V 3.4 in linear mode with 60 Hz laser intensity (200 shots) on Microflex with reflectron MALDI mass spectrometer (Bruker Daltonics). Mass was analyzed in flexAnalysis V3.4 software of Bruker Daltonics by comparing it with calibrants (protein calibration kit I, Bruker Daltonics).

Supplementary method S5

Construction of the cassette to knock-out the *Lbp* gene

The beta lactamase gene (*bla*) was amplified from the vector pLEXI I ble3 (Jena Bioscience, Germany). 351 bp upstream (fragment 1) and 300 bp downstream (fragment 2) to *Lbp* gene were amplified by PCR from genomic DNA of *S. pneumoniae* (clinical isolate SPH). Master mixes for amplification each fragment consisted of: 5 µL of High fidelity buffer (Jena Bioscience), 0.5 µL of 12.5 mM dNTP, 0.6 µL of forward primer (25 µM), 0.6 µL of reverse primer (25 µM), 5 µL of DNA template (100-500 ng), 0.5 µL of high fidelity polymerase (1.25 U) and water up to 50 µL. Primer are presented in Table 1. Note that, fragment 1, *bla* gene and fragment 2 were fused together with overlap extension PCR (OE-PCR). Primers with overhang were designed to facilitate fusion of 3 fragments to obtain fusion construct ***Fragment 1- bla-Fragment 2*** (details are in **supplementary Fig. S11, panel A)**. Cycling conditions for all fragments were: [95°C for 2 min., 35 cycles of (95°C-2 min., 55°C-30 sec, 68°C- 1 min.), 68°C- 2 min.]. Amplicons were excised from 0.7% borax agarose gel and purified with NucleoSpin (Macherey-Nagel, Germany).

All three purified fragments were mixed in equimolar concentration (5 nM) in the presence of 2.5 µL of Taq buffer (Jena Bioscience), 0.5 µL of dNTP, 0.2 µL of Taq polymerase (1 U, Jena Bioscience) and water up to 25 µL. Cycling condition for first fusion step was: [95°C-2 min, 11 cycles of (95°C-2 sec, 55°C-30 sec, 72°C-2 min), 72°C-10 min]. PCR product was column purified and subjected for second OE-PCR.

Master mix of second OE-PCR contained: 2.5 µL of amplified product from first OE-PCR, 2.5 µL of Taq buffer, 0.5 µL of dNTP, 0.4 µL of each primer KO Lbp-F1 F and KO-Lbp F2 R (25 µM), 0.2 µL of Taq polymerase and water up to 25 µL. Cycling conditions were: [95°C-2 min, 31 cycles of (95°C-2 sec, 55°C-30 sec, 72°C-2 min), 72°C-10 min]. Amplicon of the fused fragment was gel purified purified (Macherey-Nagel, Germany) and used for transformation of *S. pneumoniae*.

Please note that, *fragment 1-bla-fragment 2* fused cassette is designed to replace Lbp gene via homologous crossover.

Supplementary method S6

Transformation of *S. pneumoniae*

S. pneumoniae was grown on Columbia agar blood base containing 5% (v/v) sheep blood and single isolated colony was propagated in liquid medium as described in our previous publication[13] till OD 0.5. Culture was centrifuges, pellet was washed three times with 500 µL of 0.3 M sucrose. Finally, bacteria were resuspended in 100 µL of 0.3 M sucrose and transferred into the tube containing 200 ng of dried cassette (produced with OE-PCR). Mixture was incubated for 10 minutes incubation at room temperature and 1 min on ice. Electroporation conditions were: 2 mm gap cuvette, 2.5 kV, 200 Ω and 25 µF in Gene Pulser Xcell (Bio-Rad). Bacteria were resuspended immediately in 1 mL of Todd-Hewitt broth (Oxoid, UK), incubated 6 hours at 37°C in 5% CO2 atmosphere and then transferred into 5 mL of Todd-Hewitt broth medium containing carbenicillin (concentration 12.5 µg/mL). After 12 hours 150 µL of culture was plated on Columbia agar blood base agar containing 5% (v/v) sheep blood and carbenicillin (12.5 µg/mL), and incubated at 37°C in 5% CO2 until single isolated colonies were appeared (48 hours).

Single isolated colony (clone) was picked, resuspended in 100 µL of Todd-Hewitt broth. 50 µL from suspension was inoculated in 5 ml of Todd-Hewitt broth containing carbenicillin. Culture was grown 24 hours as described above and glycerol stocks were prepared. Remaining 50 µL of the suspension was used to isolate genomic DNA (95°C for 10 min heating). Deletion of *Lbp* was confirmed by PCR using KO-Lbp F and KO-Lbp R primers as described above. Insertion of *bla* gene (and deletion of Lbp) was confirmed by sequencing.

1 Káňová, E. *et al.* Transcriptome analysis of human brain microvascular endothelial cells response to *Neisseria meningitidis* and its antigen MafA using RNA-seq. *Scientific Reports* **9**, 1-16 (2019).
